# WNT5A promotes the metastasis of esophageal squamous cell carcinoma by activating the HDAC7/SNAIL signaling pathway

**DOI:** 10.1038/s41419-022-04901-x

**Published:** 2022-05-20

**Authors:** Yingtong Feng, Zhiqiang Ma, Minghong Pan, Liqun Xu, Junjun Feng, Yimeng Zhang, Changjian Shao, Kai Guo, Hongtao Duan, Yujing Zhang, Yuxi Zhang, Jiao Zhang, Di Lu, Xiaoya Ren, Jing Han, Xiaofei Li, Xiaolong Yan

**Affiliations:** 1 Department of Thoracic Surgery, Tangdu Hospital, The Air Force Medical University, 1 Xinsi Road, Xi’an, 710038 China; 2 Department of Cardiothoracic Surgery, the 71th Group Army Hospital of PLA/the Affiliated Huaihai Hospital of Xuzhou Medical University, 236 Tongshan Road, Xuzhou, 221004 China; 3 Department of Medical Oncology, Senior Department of Oncology, Chinese PLA General Hospital, The Fifth Medical Center, 28 Fuxing Road, Beijing, 100853 China; 4 Department of Aerospace Medicine, The Air Force Medical University, 169 Changle West Road, Xi’an, 710032 China; 5 Department of Human Resource Management, the 71th Group Army Hospital of PLA/ the Affiliated Huaihai Hospital of Xuzhou Medical University, 236 Tongshan Road, Xuzhou, 221004 China; 6 Department of Ophthalmology, Tangdu Hospital, The Air Force Medical University, 1 Xinsi Road, Xi’an, 710038 China; 7 Department of Thoracic Surgery, Shaanxi Provincial People’s Hospital, Xi’an, 710068 China; 8 Department of Cardiovascular Surgery, Xijing Hospital, The Air Force Medical University, 15 Changle West Road, Xi’an, 710032 China; 9 Department of Thoracic Surgery, Xi’an International Medical Center Hospital, 777 Xitai Road, Xi’an, 710100 China

**Keywords:** Oesophageal cancer, Metastasis, Epigenetics

## Abstract

Esophageal squamous cell carcinoma (ESCC) is one of the most common cancers worldwide, with high incidence and mortality rates and low survival rates. However, the detailed molecular mechanism of ESCC progression remains unclear. Here, we first showed significantly higher WNT5A and SNAIL expression in ESCC samples than in corresponding paracancerous samples. High WNT5A and SNAIL expression levels correlated positively with lymphatic metastasis and poor prognosis for patients with ESCC based on immunohistochemical (IHC) staining of 145 paired ESCC samples. Spearman’s correlation analyses confirmed the strong positive correlation between WNT5A and SNAIL expression, and patients with ESCC presenting coexpression of WNT5A and SNAIL had the worst prognosis. Then, we verified that the upregulation of WNT5A promoted ESCC cell metastasis in vivo and in vitro, suggesting that WNT5A might be a promising therapeutic target for the prevention of ESCC. Furthermore, WNT5A overexpression induced the epithelial-mesenchymal transition via histone deacetylase 7 (HDAC7) upregulation, and HDAC7 silencing significantly reversed WNT5A-induced SNAIL upregulation and ESCC cell metastasis. In addition, we used HDAC7 inhibitors (SAHA and TMP269) to further confirm that HDAC7 participates in WNT5A-mediated carcinogenesis. Based on these results, HDAC7 is involved in WNT5A-mediated ESCC progression, and approaches targeting WNT5A and HDAC7 might be potential therapeutic strategies for ESCC.

## Introduction

Esophageal squamous cell carcinoma (ESCC) is a malignant tumor of esophageal epithelial cells, accounting for approximately 90% of esophageal cancers, and is the eighth most common cancer in the world [1]. WNT proteins are a large family of secreted molecules rich in cysteine that regulate various cellular processes, such as proliferation, differentiation, apoptosis, and migration [2]. At least 19 members of the mammalian WNT gene family have been identified and divided into two different classes according to their transforming potential [3]. WNT5A belongs to the nontransformed category and elicits β-catenin-independent noncanonical signaling by binding to different receptors or coreceptor complexes, such as Frizzled, receptor related to tyrosine kinases (RYK), receptor tyrosine kinase-like orphan receptor-1/2 (ROR1/2), low-density lipoprotein receptor-related protein 5/6 (LRP5/6) and CD146, thus playing a crucial role in normal development [4–6]. WNT5A has been reported to possess anticancer or carcinogenic activities, depending on the tumor type and stage, and regulates various cellular responses, including convergent elongation, cell polarity, inhibition of β-catenin signaling-induced cell movement, and axon rejection [7, 8]. WNT5A promotes epithelial-mesenchymal transition (EMT)-like changes in breast cancer, gastric cancer, pancreatic cancer, ovarian cancer, and malignant melanoma [9–12] The transcription factor SNAIL is considered a key stimulator of the EMT. Many studies have shown that SNAIL is the convergence point of multiple signaling pathways, including WNT signaling, which ultimately leads to the EMT [10]. Furthermore, WNT5A is a potential prognostic marker for cancer progression. However, WNT5A expression and its role in ESCC remain unclear.

Histone deacetylases (HDACs) IIa are chromatin-modifying enzymes that can regulate many aspects of cell biology, including proliferation, differentiation, autophagy, apoptosis, migration, and angiogenesis [13–16]. HDACs consist of 18 subtypes and have been identified and classified into four categories [14]. Abnormal expression of HDACs contributes to the development and progression of cancer [16–19]. HDAC7 is a member of the HDAC IIa family and is related to many transcription factors and corepressor factors through its deacetylase activity and deacetylase-independent mechanisms, including a myocyte-enhancer family of proteins 2 A/C/D (MEF2A/C/D), signal transducer and activator of transcription 3 (STAT3), hypoxia-inducible factor-1 alpha (HIF-1α), estrogen receptor alpha (ERα), Forkhead box P3 (FOXP3), Kruppel-like factor 4 (KLF4), Runt-related transcription factor 2 (RUNX2), Forkhead box A1 (FOXA1), nuclear receptor corepressor 1 (NCOR1), and NCOR2/silencing mediator of thyroid hormone receptor (SMRT) [20]. Several studies have shown that HDAC7 is dysregulated in many cancers, including nasopharyngeal carcinoma, gastric cancer, and glioma; this dysregulation is associated with metastasis and a poor prognosis and is the main target of several HDAC inhibitors (HDACis) [21–23]. However, whether HDAC7 overexpression promotes ESCC progression remains unclear.

In the present study, we first showed that WNT5A expression was associated with a poor prognosis and promoted the lymphatic metastasis of ESCC. WNT5A overexpression induced the EMT by upregulating SNAIL expression and downregulating E-cadherin, which was associated with tumor invasion and metastasis. All these results suggested that WNT5A might be a promising therapeutic target for the prevention of ESCC progression. Then, we verified that the upregulation of WNT5A promoted HDAC7 expression. Furthermore, we investigated whether HDAC7 was involved in WNT5A-mediated ESCC progression. HDAC7 silencing or HDACis significantly reversed WNT5A-induced SNAIL upregulation and ESCC cell invasion and metastasis, highlighting the potential value of targeting the WNT5A/HDAC7/SNAIL signaling pathway to disrupt the pathophysiological process of ESCC.

## Materials and methods

### Cell culture and lentivirus infection

Human ESCC cell lines (EC109, EC9706, TE1, KYSE30, KYSE140, KYSE150, KYSE410, KYSE450, and KYSE510) were obtained from Hunan Fenghui Biotechnology Co., Ltd. (Changsha, China) or Shanghai Fuxiang Biological Technology Co., Ltd. (Shanghai, China) and cultured in DMEM (EC109, TE1, and KYSE30 cells) or RPMI 1640 (EC9706, KYSE140, KYSE150, KYSE410, KYSE450, and KYSE510 cells) (Gibco, NY, USA) supplemented with 10% fetal bovine serum (FBS, Gibco, NY, USA) and penicillin-streptomycin solution (100 units/ml) (Solarbio, Beijing, China). WNT5A, HDAC7, and empty vector lentiviruses were obtained from GeneChem (Shanghai, China), and their detailed information is listed in Supplementary Tables 3, 4. EC109, EC9706, KYSE30, and KYSE450 cells were infected with lentiviruses according to the protocol provided by GeneChem Corporation.

### Samples from patients with ESCC, tissue microarray, and immunohistochemistry (IHC)

This retrospective study enrolled 145 ESCC patients who underwent surgery at Tangdu Hospital between January 2012 and August 2015 and was approved by the ethics committee of the aforementioned hospital (No. TDLL-202110-02). Written informed consent was obtained from all participants before the study. None of the patients had received preoperative radiotherapy, chemotherapy, or targeted therapy, and the follow-up data were updated until August 2020 or death, whichever occurred first. The 145 pairs of ESCC tissues and corresponding paracancerous tissues were prepared into paraffin-embedded tissue microarray chips. IHC staining was performed on the tissue microarray chips using anti-WNT5A (1:50, 55184-1-AP, Proteintech) and anti-SNAIL (1:200, XT4351, Dingguo Bio) primary antibodies according to previously described protocols [24]. The immunostaining intensity was scored as 0 (negative), 1 (weak), 2 (moderate), or 3 (strong), and the proportion of positively stained cells was scored as 0 (<5%), 1 (6–25%), 2 (26–50%), 3 (51–75%), or 4 (>75%) (Supplementary Fig. S3C–E). The two scores were multiplied to produce the total score. ESCC samples were stratified into low or high WNT5A/SNAIL expression groups according to their respective average score.

### Wound healing assay

Cells were seeded into six-well plates with a routine culture medium containing 10% FBS. A scratch was generated with a 200-μl pipette tip after the cells reached 90–100% confluence. Then, each well was rinsed three times with 1× PBS, a medium containing 3% FBS was added, and images were immediately captured. The gaps between the wound edges were monitored and photographed under a microscope after 48 h.

### Transwell cell migration and invasion assays

Transwell assays were performed to detect cell migration and invasion. First, cells were cultured in serum-free media (SFM) for 24 h and then seeded in the upper chamber (Costar, Corning, NY, USA) coated with Matrigel (for invasion assays) or uncoated (for migration assays). The upper chamber was filled with 200 μl SFM, and the lower chamber was filled with the same medium supplemented with 10% FBS. After 24 h, the migrated or invaded cells attached to the lower surface of the upper chamber were fixed with formalin, stained with 0.1% crystal violet, photographed, and counted using ImageJ software.

### Western blotting

Western blotting was performed following procedures described previously [25]. The following primary antibodies were used: anti-WNT5A (1:1000, bs-1948R, BIOSS), anti-FLAG (1:1000, 66008-3-Ig, Proteintech), anti-HDAC7 (1:1000, #33418, CST), anti-SNAIL (1:1000, 13099-1-AP, Proteintech), anti-E-cadherin (1:1000, #14472, CST), anti-SLUG (1:1000, #9585, CST), anti-vimentin (1:1000, 10366-1-AP, Proteintech), anti-TWIST1 (1:1000, 25465-1-AP, Proteintech), anti-HDAC1 (1:1000, #5356, CST), anti-HDAC2 (1:1000, #5113, CST), anti-HDAC3 (1:1000, #3949, CST), anti-HDAC4 (1:1000, 17449-1-AP, Proteintech), anti-HDAC5 (1:1000, 16166-1-AP, Proteintech), anti-HDAC6 (1:1000, 12834-1-AP, Proteintech), anti-HDAC8 (1:1000, E-AB-14127, Elabscience), anti-HDAC9 (1:1000, 67364-1-Ig, Proteintech), anti-HDAC10 (1:1000, 24913-1-AP, Proteintech), anti-HDAC11 (0.3 µg/mL, PA5-79351, Thermo), and anti-β-actin (1:1000, #3700, CST). The secondary antibodies used were HRP-linked anti-IgG antibodies (1:5000, Zhongshan Company, Beijing, China).

### Nude mouse lung metastasis assay

Animal experiments were approved by the Animal Care Committee of Air Force Medical University (No. IACUC-20210390). Male athymic nude mice (6–8 weeks) were obtained from the Laboratory Animal Center of the aforementioned university. The animals were divided into different groups (*n* = 5 per group) and injected with various cells (7 × 10^6^ in 100 µL PBS) through the tail vein, and their body weights were measured every 3 days. After 7 weeks, the mice were imaged using a small animal in vivo imaging system (IVIS LuminaX5, PerkinElmer, USA). Then, the mice were sacrificed, their lungs were dissected for a standard histological examination, and the lung metastatic nodules of each mouse were counted for statistical analyses.

### Statistical analyses

SPSS 23.0 was used to analyze the data. The student’s *t*-test was used to compare the quantitative data between two groups. The χ^2^ test or Fisher’s exact test was used to assess the relationships between WNT5A/SNAIL expression and clinicopathological characteristics of patients with ESCC. Kaplan–Meier plots and log-rank tests were used to assess overall survival rates. Univariate and multivariate survival analyses were conducted using the Cox proportional hazards model. Data are presented as the means ± SD. A value of *P* < 0.05 (two-tailed) was considered to indicate significance.

## Results

### Elevated expression of WNT5A in cancer tissues correlates with a poor prognosis for patients with ESCC

**Fig. 1:**
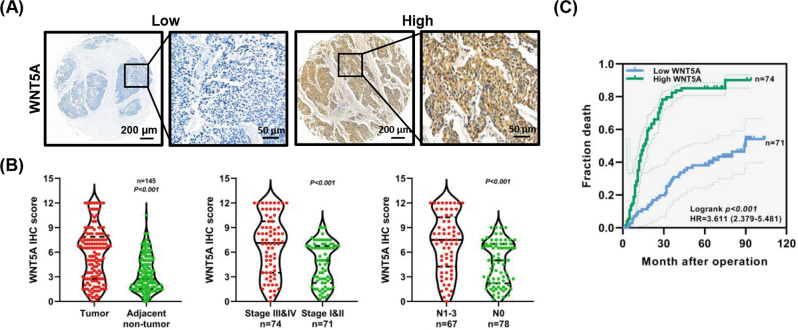
High WNT5A expression in ESCC tissues is correlated with a poor prognosis. **A** Representative IHC images showing WNT5A expression in ESCC tissues. Scale bars, 200 and 50 μm (inset). **B** Statistical analysis of WNT5A expression in 145 patients with ESCC based on the IHC results from the tissue microarray. **C** Kaplan–Meier survival analysis of 145 patients with ESCC presenting high/low WNT5A expression.

The WNT5A protein level was detected using IHC staining in a tissue microarray containing 145 paired ESCC tumor-normal tissues (Fig. 1A). WNT5A was expressed at significantly higher levels in ESCC tissues than in the adjacent noncancerous samples (*P* < 0.001, Fig. 1B). Moreover, patients with ESCC presenting lymph node metastasis (N1–3) and a high American Joint Committee on Cancer (AJCC) seventh stage (stages III/IV) had higher WNT5A expression than those without lymph node metastasis (N0) and with a low seventh edition AJCC stage (stages I/II) (*P* < 0.001, Fig. 1B). Furthermore, elevated WNT5A expression was positively correlated with lymphatic invasion and the seventh edition AJCC stage (*P* < 0.05, Table 1).

**Table 1:** Correlations of WNT5A and SNAIL expression with the clinicopathological characteristics of patients with ESCC.

Clinicopathological variables	*n*	WNT5A expression			SNAIL expression		
		Low	High	*P* value	Low	High	*P* value
**Age**				0.480			0.795
<60	45	24	21		21	24	
≥60	100	47	53		49	51	
**Gender**				0.752			0.289
Female	26	12	14		15	11	
Male	119	59	60		55	64	
**Smoking history**				0.293			0.398
Never	57	31	26		30	27	
Ever	88	40	48		40	48	
**Tumor size**				0.724			0.127
<5 cm	49	25	24		28	21	
≥5 cm	96	46	50		42	54	
**TNM stages**				0.038			0.025
I–II	71	41	30		41	30	
III–IV	74	30	44		29	45	
**Tumor invasion**				0.832			0.316
T1–T2	13	6	7		8	5	
T3–T4	132	65	67		62	70	
**Lymphatic invasion**				0.023			0.005
N0	78	45	33		46	32	
N1–N3	67	26	41		24	43	
**Differentiation**				0.727			0.533
Well and moderate	128	62	66		63	65	
Poorly and not	17	9	8		7	10	

The results of the Kaplan–Meier analysis showed that patients with ESCC presenting high WNT5A expression experienced shorter overall survival (log-rank *P* < 0.001, Fig. 1C). Likewise, the 3- and 5-year cumulative survival rates for ESCC patients with high WNT5A expression (17.6 and 14.9%, respectively) were much lower than those for patients with low WNT5A expression (70.4 and 62.0%, respectively) (Supplementary Table 1). Furthermore, Cox survival analyses suggested that WNT5A was an independent prognostic factor for patients with ESCC (HR = 2.310, 95% CI: 1.423–3.748, *P* = 0.001, Supplementary Table 2). These results indicated that WNT5A expression was associated with a poor prognosis for patients with ESCC and that the promotion of lymph node metastasis might be an important factor.

### WNT5A overexpression activates SNAIL and patients with ESCC coexpressing these molecules have the poorest prognosis

**Fig. 2:**
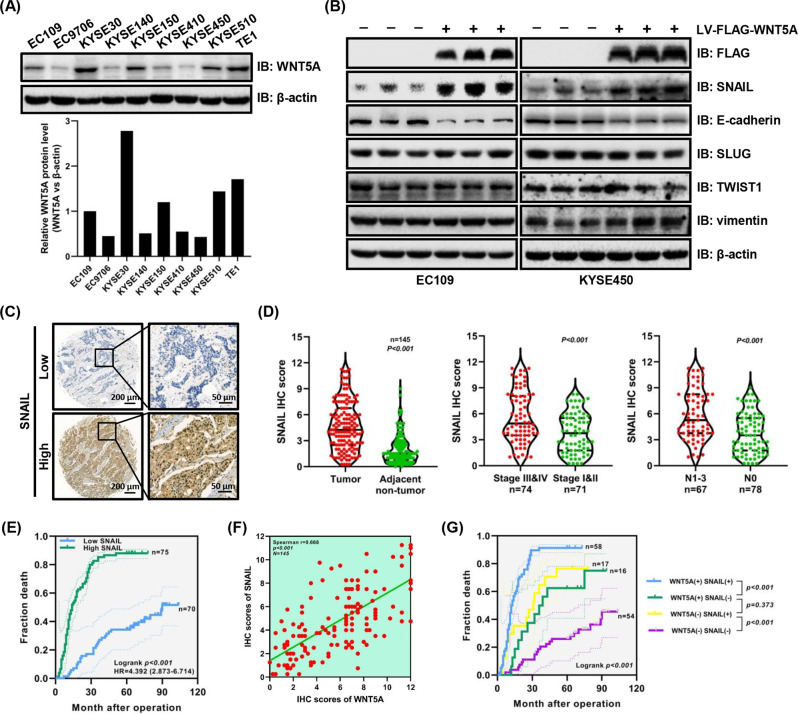
SNAIL levels are significantly upregulated with WNT5A overexpression and correlate with a poor prognosis. **A** Western blots showing WNT5A levels in various ESCC cells. **B** Representative western blots showing the levels of FLAG-WNT5A, SNAIL, E-cadherin, SLUG, TWIST1, and vimentin in WNT5A-overexpressing cells. β-actin was used as an internal control. **C** Representative IHC images showing SNAIL expression in ESCC tissues. Scale bars, 200 and 50 μm (inset). **D** Statistical analysis of SNAIL expression in 145 patients with ESCC based on the tissue microarray IHC results. **E** Kaplan–Meier survival analysis of 145 patients with ESCC presenting high/low SNAIL expression. **F** Correlation analysis of WNT5A and HDAC7 expression in ESCC tissues. **G** Comprehensive Kaplan–Meier survival analysis of 145 patients with ESCC presenting high/low WNT5A and SNAIL expression. LV lentivirus.

The EMT is closely related to tumor invasion and metastasis [26]. To study the correlation between WNT5A and EMT in ESCC, we assessed WNT5A expression levels in multiple ESCC cell lines and found that WNT5A expression levels in EC109, EC9706, KYSE140, KYSE410, and KYSE450 cells were relative low, whereas the expression levels in KYSE30, KYSE510, and TE1 cells were relatively high (Fig. 2A). Then, we established stable WNT5A-overexpressing EC109/KYSE450 cell lines and WNT5A knockdown KYSE30 cell lines using LV-WNT5A (WNT5A OE) or LV-WNT5A-RNAi (shWNT5A) lentivirus. Afterward, we examined the expression of EMT-associated proteins by western blotting and found that WNT5A overexpression significantly increased the expression of the EMT facilitator SNAIL and decreased the expression of the epithelial marker E-cadherin. The opposite trend was observed in the WNT5A knockdown group. However, the levels of other EMT biomarkers, such as SLUG, TWIST1, and vimentin, did not change (Fig. 2B and Supplementary Fig. S1B–D). In addition, we also determined the SNAIL expression levels in the nine ESCC cell lines and found the trends of WNT5A and SNAIL expression levels were consistent in the majority of ESCC cell lines (Fig. 2A and Supplementary Fig. S1A). These results revealed that WNT5A overexpression might activate SNAIL and promote the EMT in ESCC cells, which plays an important role in tumor metastasis.

After identifying the relationship between WNT5A and SNAIL, we further detected SNAIL expression by performing an IHC analysis of a tissue array containing 145 paired tumor-normal samples (Fig. 2C). SNAIL expression in ESCC samples was higher than that in adjacent noncancerous samples (*P* < 0.001, Fig. 2D). Moreover, ESCC patients presenting with lymph node metastasis (N1–3) and a high seventh edition AJCC stage (stages III/IV) exhibited higher expression of SNAIL than those without lymph node metastasis (N0) and with a low seventh edition AJCC stage (stages I/II) (*P* < 0.001, Fig. 2D). High SNAIL expression was positively correlated with lymphatic invasion and the seventh edition AJCC stage (*P* < 0.05, Table 1). Furthermore, Kaplan–Meier survival curves indicated that ESCC patients presenting with high SNAIL expression had a worse prognosis than those with low expression (log-rank *P* < 0.001, Fig. 2E). The 3- and 5-year cumulative survival rates for ESCC patients exhibiting high SNAIL expression (14.7 and 12.0%, respectively) were much lower than those for ESCC patients presenting low SNAIL expression (74.3 and 65.7%, respectively) (Supplementary Table 1). Moreover, Cox survival analyses suggested that SNAIL was an independent prognostic factor for patients with ESCC (HR = 3.596, 95% CI: 2.145–6.030, *P* < 0.001, Supplementary Table 2). Intriguingly, the correlation analysis showed a significant correlation between WNT5A and SNAIL expression in ESCC tissues (*P* < 0.001, Fig. 2F). Furthermore, Kaplan–Meier survival curves showed that patients with high WNT5A and high SNAIL expression had the worst prognosis, whereas patients with low WNT5A and low SNAIL expression had the best prognosis (Fig. 2G). These results suggested that WNT5A facilitated ESCC lymph node metastasis by inducing SNAIL expression.

### WNT5A overexpression promotes ESCC progression

**Fig. 3:**
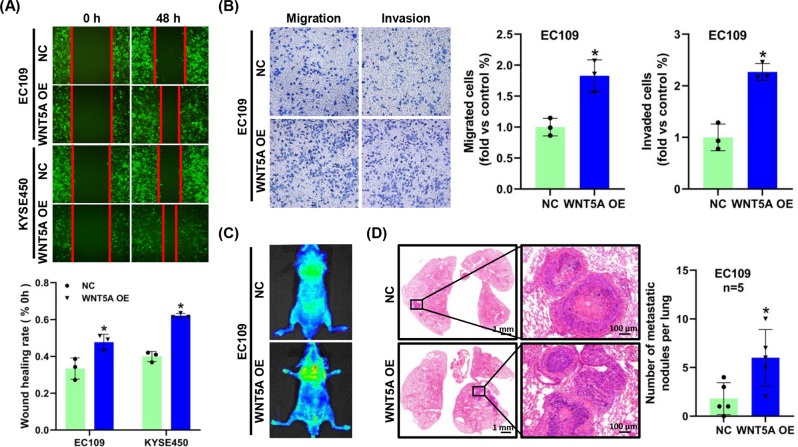
WNT5A overexpression facilitates the migration, invasion, and metastasis of ESCC cells. **A**, **B** Representative images and statistical analysis of wound healing assays and Transwell migration/invasion assays using EC109 or KYSE450 cells overexpressing WNT5A. The migratory ability in wound healing assays was expressed as the mean scratch area. The initial scratch area (0 h) was set to 100%. **C** Representative fluorescence images of nude mice 7 weeks after the tail vein injection. **D** Representative images of HE staining of lung samples and statistical analysis of the metastatic nodules per lung from the indicated groups (*n* = 5 per group). Scale bars, 1 mm and 100 μm (inset). *P < 0.05 compared with the NC group.

We assessed the changes in the metastatic phenotype of ESCC cells transfected with the WNT5A lentivirus to study the role of WNT5A in ESCC progression in vitro. Compared with the control groups, WNT5A overexpression significantly increased the migration and invasion of both EC109 and KYSE450 cells, whereas WNT5A knockdown decreased these functions in KYSE30 cells, which was validated by the Transwell and wound healing assays (*P* < 0.05, Fig. 3A, B and Supplementary Fig. S1E, F). We then performed a tail vein metastasis experiment in nude mice to investigate the role of WNT5A in promoting ESCC cell metastasis and found that the upregulation of WNT5A increased the incidence of lung metastasis (*P* < 0.05, Fig. 3C, D).

### HDAC7 promotes ESCC cell metastasis and is positively regulated by WNT5A signaling

**Fig. 4:**
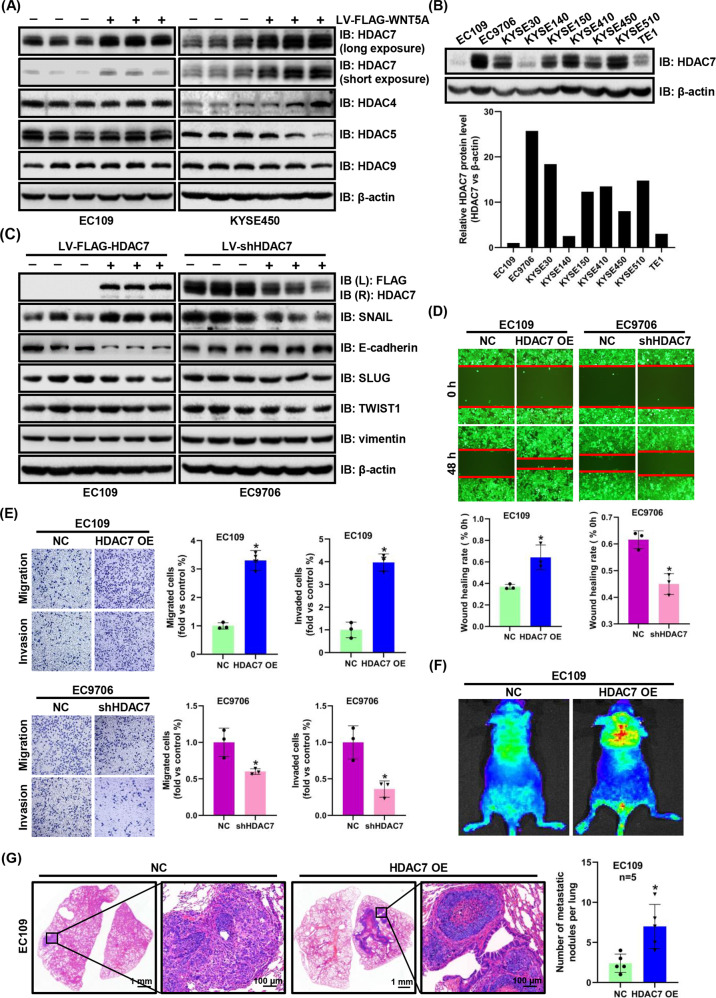
HDAC7 expression is significantly upregulated by WNT5A overexpression and HDAC7 overexpression facilitates ESCC metastasis in vitro and in vivo. **A** Representative western blots showing the levels of HDAC7, HDAC4, HDAC5, and HDAC9 in WNT5A-overexpressing cells. **B** Western blots showing HDAC7 levels in various ESCC cells. **C** Representative western blots showing FLAG-HDAC7, HDAC7, SNAIL, E-cadherin, SLUG, TWIST1, and vimentin levels in HDAC7-overexpressing or HDAC7 knockdown ESCC cells. **D**, **E** Representative images and statistical analysis of wound healing assays and Transwell migration/invasion assays using HDAC7 OE and shHDAC7 ESCC cells. The migratory ability in wound healing assays was expressed as the mean scratch area. The initial scratch area (0 h) was set to 100%. **F** Representative fluorescence images of nude mice 7 weeks after the tail vein injection. **G** Representative images of HE staining of lung samples and statistical analysis of the metastatic nodules per lung (*n* = 5 per group). Scale bars, 1 mm and 100 μm (inset). β-actin was used as an internal control. *P < 0.05 compared with the NC group. LV lentivirus, L left, R right.

We subsequently examined the expression of HDACs after WNT5A overexpression or knockdown in ESCC cells using western blotting to explore the underlying molecular mechanisms by which WNT5A overexpression promotes ESCC metastasis and found that only the expression of HDAC7 was upregulated or downregulated, while other HDACs showed no changes (Fig. 4A and Supplementary Fig. S2A–D). To investigate the role of HDAC7 in ESCC metastasis, we firstly assessed HDAC7 expression in multiple ESCC cell lines (Fig. 4B), and then we established two stable cell lines via lentiviral infection (HDAC7-overexpressing EC109 cell line and HDAC7 knockdown EC9706 cell line). We detected the expression of EMT-associated proteins using western blotting and found that HDAC7 overexpression significantly increased the SNAIL expression level and decreased the E-cadherin expression level; the opposite trend was observed in the HDAC7 knockdown group. Meanwhile, the levels of the other EMT biomarkers, such as SLUG, TWIST1, and vimentin, did not change (Fig. 4C and Supplementary Fig. S2E). Similar to the results of the WNT5A experiments, the metastatic capacities were changed by manipulating HDAC7 expression. HDAC7 overexpression remarkably increased the migration and invasion of EC109 cells, as verified by Transwell and scratch wound assays. In contrast, downregulating HDAC7 reduced the migratory and invasive capacities of EC9706 cells (Fig. 4D, E). Similarly, we investigated the role of HDAC7 in promoting ESCC metastasis by performing a nude mouse lung metastasis assay and found that overexpression of HDAC7 increased the incidence of lung metastasis, whereas loss of HDAC7 exerted the opposite effect (Fig. 4F, G and Supplementary Fig. S2G, H).

### HDAC7 is involved in WNT5A-mediated ESCC progression

**Fig. 5:**
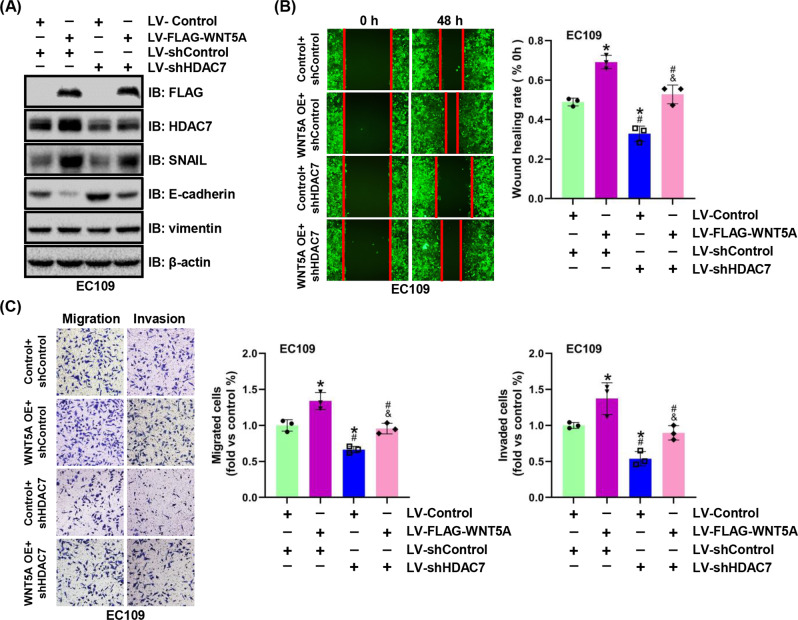
HDAC7 is essential for WNT5A-mediated ESCC cell migration and invasion. **A** Representative western blots showing the levels of FLAG-WNT5A, HDAC7, SNAIL, E-cadherin, and vimentin in EC109 cells with WNT5A overexpression and/or HDAC7 knockdown. β-actin was used as an internal control. **B**, **C** Representative images and statistical analysis of wound healing assays and Transwell migration/invasion assays in the indicated ESCC cells. The migratory ability in wound healing assays was expressed as the mean scratch area. The initial scratch area (0 h) was set to 100%. *P < 0.05 compared with the LV-Control + shControl group, ^#^*P* < 0.05 compared with the LV-FLAG-WNT5A + shControl group, and ^&^*P* < 0.05 compared with the LV-Control + shHDAC7 group. LV lentivirus.

We examined the effect of HDAC7 downregulation by infecting EC109 cells overexpressing WNT5A with the HDAC7-RNAi lentivirus to confirm the involvement of HDAC7 in WNT5A-mediated ESCC progression. Using western blotting, the according changes of HDAC7 and FLAG-WNT5A expressions were verified, and HDAC7 knockdown reversed WNT5A overexpression induced changes in the levels of EMT biomarkers by decreasing SNAIL and increasing E-cadherin levels (Fig. 5A and Supplementary Fig. S3A). Moreover, the increased migration and invasion induced by WNT5A were partially inhibited by HDAC7 knockdown, as evidenced by the results of the scratch wound assay and Transwell assay (Figs. 5B, C). Furthermore, the nude mouse lung metastasis assay revealed that the WNT5A-mediated increase in lung metastasis was also attenuated by HDAC7 knockdown (Supplementary Fig. S3B). In summary, these results suggest that HDAC7 is implicated in WNT5A-mediated ESCC progression and that HDAC7 silencing partially attenuates the migration, invasion, and metastasis induced by WNT5A.

### Targeted inhibition of HDAC7 inhibits WNT5A-induced ESCC progression

**Fig. 6:**
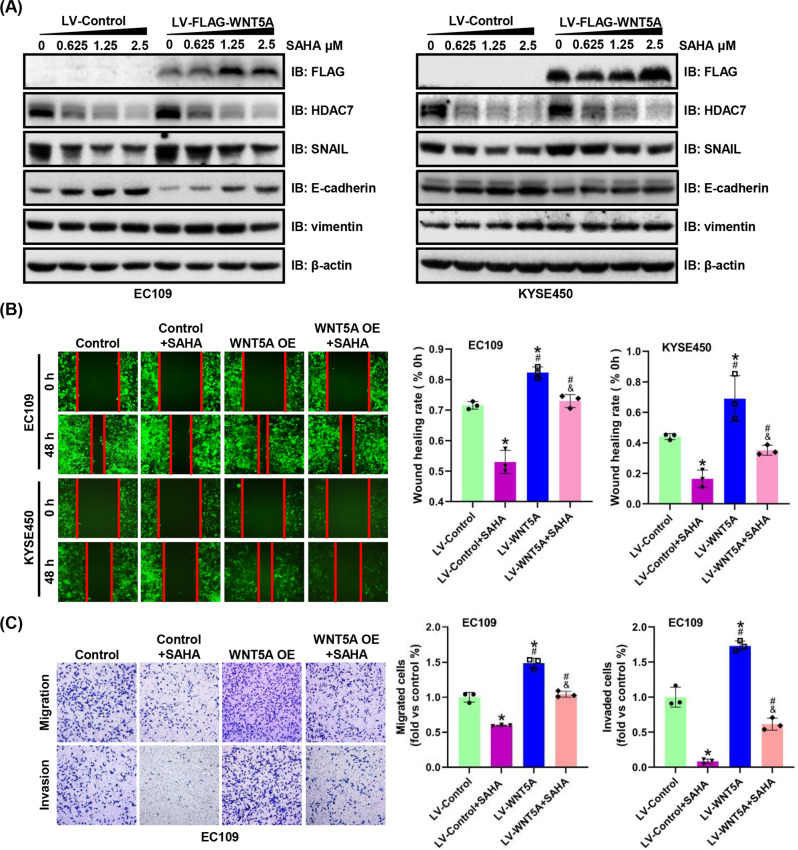
SAHA inhibits WNT5A-mediated ESCC cell migration and invasion. **A** Representative western blots showing the levels of FLAG-WNT5A, HDAC7, SNAIL, E-cadherin, and vimentin in WNT5A-overexpressing EC109 and KYSE450 cells after treatment with SAHA for 48 h. β-actin was used as an internal control. **B**, **C** Representative images and statistical analysis of wound healing assays and Transwell migration/invasion assays using WNT5A-overexpressing ESCC cells after treatment with SAHA (1.25 μM) for 48 h. The migratory ability in wound healing assays was expressed as the mean scratch area. The initial scratch area (0 h) was set to 100%. *P < 0.05 compared with the LV-Control group, ^#^*P* < 0.05 compared with the LV-Control + SAHA group, and ^&^*P* < 0.05 compared with the LV-WNT5A group. LV lentivirus.

**Fig. 7:**
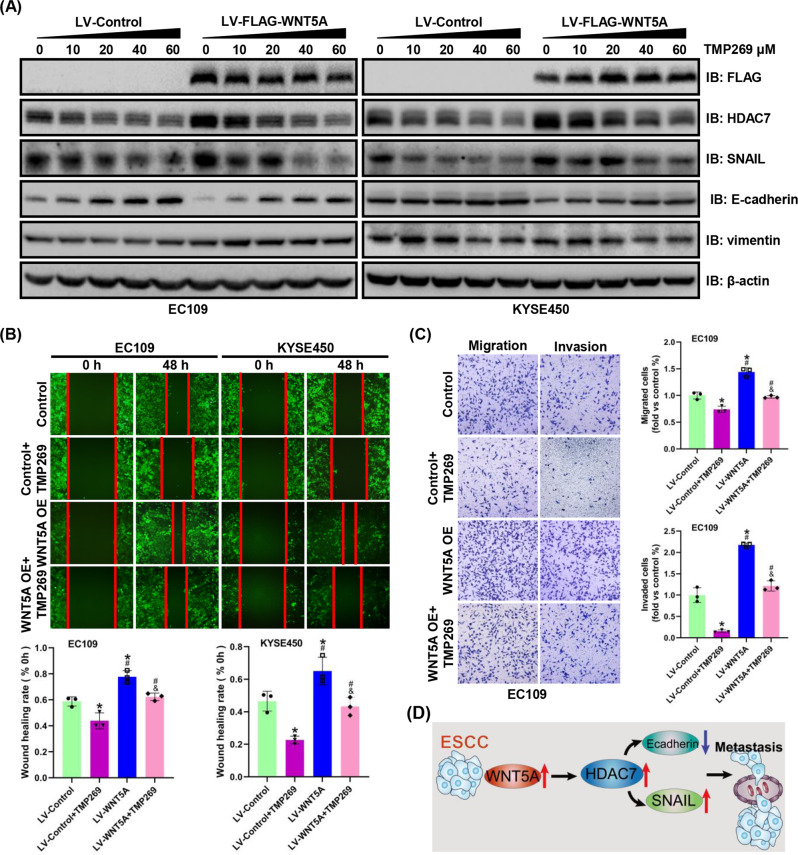
TMP269 attenuates WNT5A-mediated ESCC cell migration and invasion. **A** Representative western blots showing the levels of FLAG-WNT5A, HDAC7, SNAIL, E-cadherin, and vimentin in WNT5A-overexpressing EC109 and KYSE450 cells after treatment with TMP269 for 48 h. β-actin was used as an internal control. **B**, **C** Representative images and statistical analysis of wound healing assays and Transwell migration/invasion assays using WNT5A-overexpressing ESCC cells after treatment with TMP269 (20 μM) for 48 h. The migratory ability in wound healing assays was expressed as the mean scratch area. The initial scratch area (0 h) was set to 100%. *P < 0.05 compared with the LV-Control group, ^#^*P* < 0.05 compared with the LV-Control + TMP269 group, and ^&^*P* < 0.05 compared with the LV-WNT5A group. LV lentivirus. **D** Schematic diagram of the mechanism by which WNT5A promotes tumor metastasis by activating the HDAC7/SNAIL signaling pathway in ESCC.

To further confirm that HDAC7 participates in WNT5A-mediated ESCC progression, we used HDAC7 inhibitors (SAHA and TMP269) in our experiments. The WNT5A-overexpressing and control EC109/KYSE450 cell lines were treated with SAHA or TMP269 at various concentrations for 48 h. Notably, in both cell lines, the changes of HDAC7, SNAIL, and E-cadherin expression induced by WNT5A overexpression were inhibited by SAHA or TMP269 in a dose-dependent manner, whereas the level of the vimentin protein did not change (Figs. 6A, 7A). Furthermore, the migratory and invasive capacities were significantly restrained, and the WNT5A-induced increases in migration and invasion were attenuated by SAHA or TMP269 (Figs. 6B, C, 7B, C). Therefore, WNT5A-mediated ESCC progression is partially reversed by HDAC7 inhibition.

## Discussion

ESCC is characterized by late diagnosis, metastasis, treatment resistance, and frequent recurrence, and the underlying molecular mechanism remains elusive and warrants further investigation [27]. In the present study, our IHC analysis revealed significantly higher WNT5A expression in ESCC tissues than in adjacent noncancerous tissues and that high WNT5A expression was related to a high TNM and N stage. Additionally, the cumulative survival rate results indicated that high WNT5A expression was associated with shorter overall survival of patients with ESCC. The multivariate Cox survival analysis suggested that WNT5A was a negative prognostic factor for patients with ESCC.

WNT5A, a typical WNT protein involved in an independent branch of β-catenin signaling, is highly conserved among species and plays key roles in embryonic development, internal homeostasis, and pathological disorders. WNT5A can stimulate the noncanonical WNT pathway and the canonical WNT signaling pathway by either activating or antagonizing it [28]. Several studies reported that WNT5A was upregulated in breast carcinomas, prostate carcinomas, and melanoma, indicating its oncogenic role in these cancers [3]. Furthermore, the loss of WNT5A reduces β-catenin/E-cadherin-mediated cell–cell adhesion [29]. However, it has been shown to exert tumor-suppressive effects on colorectal cancer, thyroid cancer, and acute lymphoblastic leukemia, especially on colon cancer [3]. WNT5A protein expression is reduced in Dukes B colon cancer tissue compared with normal tissue and is negatively correlated with the five-year survival rate, suggesting that the loss of WNT5A is a potential prognostic marker for colon cancer progression [30]. Based on these results, the tumor-suppressing or tumor-stimulating effects of WNT5A depend on the cancer type and stage. Our results indicated that a high WNT5A level was associated with shorter survival of patients with ESCC.

The EMT is the key biological event in tumor cell invasion and metastasis. Cancer cells undergoing the EMT lose cell-to-cell contact and polarity and acquire characteristics of migration and invasion [31, 32]. Recent studies have emphasized the importance of WNT5A in regulating the EMT, thus stimulating cell migration, invasion, and aggressiveness in gastric, pancreatic, ovarian, and malignant melanoma; however, the role of WNT5A in ESCC has not been previously investigated [9, 12, 33]. In the present study, the IHC analysis showed that elevated WNT5A expression was positively correlated with ESCC lymph node metastasis. Subsequent western blot analyses suggested that WNT5A overexpression significantly increased the levels of the pro-EMT protein SNAIL and decreased the expression of E-cadherin, and the opposite trend was observed in the WNT5A knockdown group. Meanwhile, the levels of the other EMT biomarkers, such as SLUG, TWIST1, and vimentin, did not change. The EMT process is usually not a binary switch. Instead, cells stably maintain one or more hybrid epithelial/mesenchymal (E/M) phenotypes, which may be more metastatic than cells in “full EMT” or “extreme mesenchymal” states [34]. Incomplete EMT processes may occur in the cells used in our study based on the expression of epithelial and mesenchymal cell markers. In addition, the pathways involved in the EMT are complex, and thus not all the levels of relevant factors will change. Furthermore, we observed that WNT5A overexpression significantly increased the migration and invasion, whereas WNT5A knockdown decreased these functions of ESCC cells. The role of WNT5A in promoting tumor metastasis was further confirmed in a nude mouse lung metastasis assay. These results indicated that metastasis and EMT might be induced by WNT5A overexpression in ESCC cells.

SNAIL, a transcriptional repressor that directly inhibits the transcription of E-cadherin, is the main regulator of the EMT [35, 36]. Emerging evidence shows that SNAIL endows tumor cells with characteristics similar to those of cancer stem cells, and a high level of SNAIL expression is correlated with tumor invasion and metastasis [37, 38]. The expression of SNAIL and E-cadherin is negatively correlated in a variety of cancers. E-cadherin downregulation is related to tumor invasiveness, metastasis, and a poor prognosis in several solid tumors [39]. SNAIL suppresses E-cadherin expression through the recruitment of the Sin3A/HDAC1/2 complex via the SNAG domain to deacetylate histones H3 and H4 [40]. Furthermore, WNT5A activates SNAIL and induces the EMT, subsequently promoting tumor metastasis in melanoma and ovarian cancer [11, 41]. Interestingly, SNAIL not only induces the EMT but also activates multiple immunosuppressive and immune resistance mechanisms [42]. According to a previous study, melanoma cells transduced with SNAIL showed typical EMT characteristics accompanied by increased mRNA expression of transforming growth factor-β (TGF-β), IL-10, and TSP1 [42]. Furthermore, PD-L1 levels have been reported to increase as cells undergo the EMT in different cancer types. TGF-β application or overexpression of SNAIL results in an increase in the activity of hallmark EMT genes and PD-L1 [43]. Our study showed high SNAIL expression in ESCC, and WNT5A significantly regulated the expression of SNAIL and E-cadherin. However, whether WNT5A promotes ESCC metastasis through a mechanism related to SNAIL-mediated immunosuppression remains unclear, which needs further study.

HDAC7 is involved in the regulation of cell proliferation, differentiation, apoptosis, and metastasis under physiological and pathological conditions [44]. Abnormal HDAC7 expression has been observed in lung cancer, stomach cancer, breast cancer, ovarian cancer, glioma, and hematological lymphoma. High HDAC7 expression is associated with migration and a poor prognosis [22]. In addition, HDAC7 is the main target of several HDACis, such as SAHA and TMP269. However, the role and underlying mechanism of HDAC7 in ESCC have not yet been studied. The relationship between WNT5A and HDAC7 has not been reported thus far. A study indicates that the oncogenic roles of WNT5A are dependent on the upregulation of HDAC6 [45]. However, we did not observe any effects of HDAC7 overexpression or knockdown on WNT5A expression in ESCC cells (data in the Supplementary Fig. S2F), suggesting that other factors mediate the function of WNT5A in ESCC, which warrants further investigation. Interestingly, both WNT5A and HDACs were recently shown to be essential for cell migration [4, 46] Therefore, we explored whether WNT5A regulated HDACs in ESCC cells and found that WNT5A clearly upregulated HDAC7 using western blotting.

Furthermore, HDACs can deacetylate nonhistone proteins, in addition to histone deacetylation, transcriptional inhibition, and heterochromatin formation in the nucleus. Abnormal modification of histone deacetylases is usually associated with cancer. The acetylation status of histones at a particular DNA regulatory sequence depends on the recruitment of histone acetyltransferases or HDACs, which are usually part of a large multiprotein complex of coactivators or corepressors, respectively [40, 47]. Previous studies reported that the SNAG domain of SNAIL was located at *CDH1*, which encodes the E-cadherin promoter and inhibits E-cadherin expression, thus regulating the metastasis of tumors by recruiting a corepressor complex containing HDAC1/2 and Sin3A [47]. CREB binding protein interacts with and acetylates the K146 and K187 sites of SNAIL, thereby preventing the formation of inhibitory complexes [48]. In addition, HDACis regulate SNAIL stability by upregulating COP9 signalosome 2 (CSN2) expression, which interacts with SNAIL, exposes its acetylation site, and inhibits SNAIL degradation by preventing its phosphorylation and ubiquitination [49]. However, acetylated PEPCK1 recruits the E3 ubiquitin-protein ligase UBR5, leading to ubiquitination and degradation [50]. Similarly, acetylation of DNA (cytosine-5)-methyltransferase 1 (DNMT1) promotes ubiquitination via the E3 ubiquitin-protein ligase UHRF1, which targets DNMT1 for proteasomal degradation [51]. Therefore, acetylation can either inhibit proteasome-dependent degradation by preventing protein ubiquitination or promote protein degradation by increasing ubiquitination. In our study, HDAC7 knockdown reversed WNT5A overexpression induced changes in the levels of EMT markers by decreasing SNAIL levels. Furthermore, our research group recently found that HDAC7 increased SNAIL protein levels by regulating fibroblast growth factor 18 (FGF18) in non-small cell lung cancer [52]. As mentioned above, we speculate that SNAIL regulates E-cadherin expression by recruiting HDAC7 to form a corepressor complex, HDAC7 deacetylates lysine residue sites on the SNAIL protein to affect its stability and regulate the expression of E-cadherin, or HDAC7 indirectly promotes SNAIL by regulating other factors, but these hypotheses require further exploration.

Our studies verified that HDAC7 expression significantly upregulated the SNAIL expression and downregulated the E-cadherin expression, as well as increased the migration and invasion of ESCC cells. The nude mouse lung metastasis assay further confirmed the promotion of tumor metastasis. Moreover, we found that HDAC7 was involved in the progression of ESCC mediated by WNT5A. HDAC7 silencing significantly reversed the WNT5A overexpression-induced migration, invasion, and metastasis of ESCC cells. In addition, we used TMP269 and SAHA, which are HDAC inhibitors, to further confirm that HDAC7 participates in WNT5A-mediated ESCC progression. Based on these results, HDAC7 is responsible for WNT5A-mediated ESCC progression.

In conclusion, our studies first confirmed that WNT5A functioned as a tumor promoter by enhancing ESCC cell metastasis. In addition, elevated WNT5A and SNAIL expression were associated with a poor prognosis for patients with ESCC and that HDAC7 upregulation was involved in WNT5A-mediated ESCC progression. Therefore, approaches targeting WNT5A and HDAC7 might be a promising therapeutic strategy for future ESCC treatment.

## Supplementary information

Supplementary information

Supplementary Figure 1

Supplementary Figure 2

Supplementary Figure 3

Supplementary Table 1

Supplementary Table 2

Supplementary Table 3.

Supplementary Table 4

Reproducibility checklist

Clinical research ethics review form

The tab of animal experimental ethical inspection

Original Data File

## Data Availability

The datasets are available from the corresponding author on reasonable request.
