# Supplementary material for: WNT5A promotes the metastasis of esophageal squamous cell carcinoma by activating the HDAC7/SNAIL signaling pathway
Source: Cell Death Dis. 2022 May 20;13(5):480. doi: 10.1038/s41419-022-04901-x (PMC9122958; doi:10.1038/s41419-022-04901-x)
Supplement: Supplementary file 1 — Supplementary information [file 41419_2022_4901_MOESM1_ESM.docx]

Supplementary Information

**WNT5A promotes the metastasis of esophageal squamous cell carcinoma by activating the HDAC7/SNAIL signaling pathway**

Manuscript ID: CDDIS-21-4520RR

**Supplementary Fig. 1-3**

**Supplementary Table 1-4**


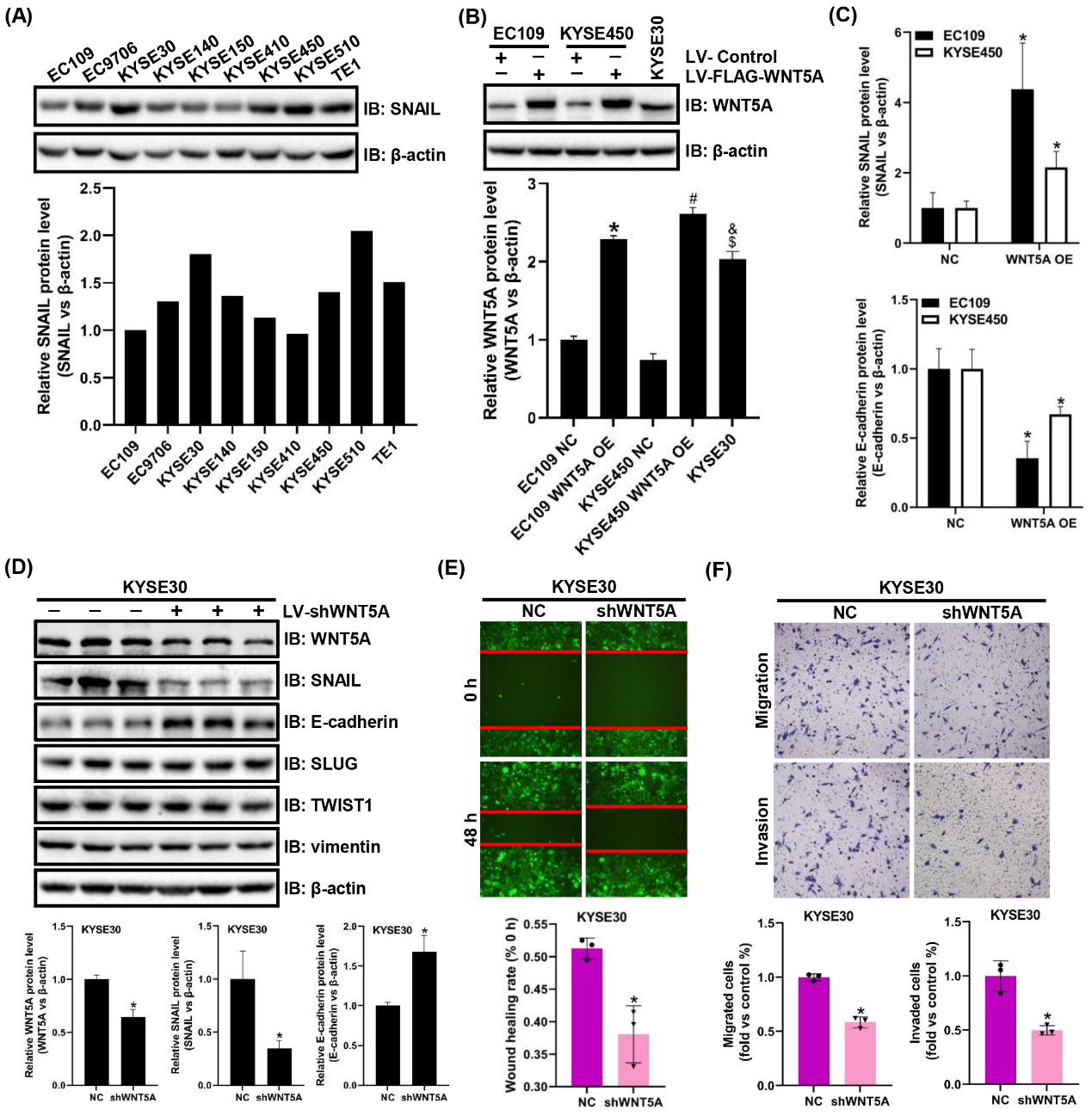


**Supplementary Fig. 1** (A) Western blot showing SNAIL levels in various ESCC cells. (B) Western blots showing WNT5A levels in WNT5A-overexpressing ESCC cells compared to the KYSE30 cell line. **P* < 0.05 compared with the EC109 NC group, ^#^*P* < 0.05 compared with the KYSE450 NC group, ^&^*P* < 0.05 compared with the EC109 WNT5A OE group, and ^$^*P* < 0.05 compared with the KYSE450 WNT5A OE group. (C) Statistical analysis of SNAIL and E-cadherin levels based on the western blotting results shown in Figure 2B. **P* < 0.05 compared with the NC group. (D) Western blots showing the levels of WNT5A, SNAIL, E-cadherin, SLUG, TWIST1, and vimentin in WNT5A knockdown KYSE30 cells. **P* < 0.05 compared with the NC group. (E-F) Representative images and statistical analyses of wound healing assays and Transwell migration/invasion assays in WNT5A knockdown KYSE30 cells. The migratory ability in wound healing assays was expressed as the mean scratch area. The initial scratch area (0 h) was set to 100%. **P* < 0.05 compared with the NC group. β-actin was used as an internal control in western blot assays. Abbreviation: LV, lentivirus.


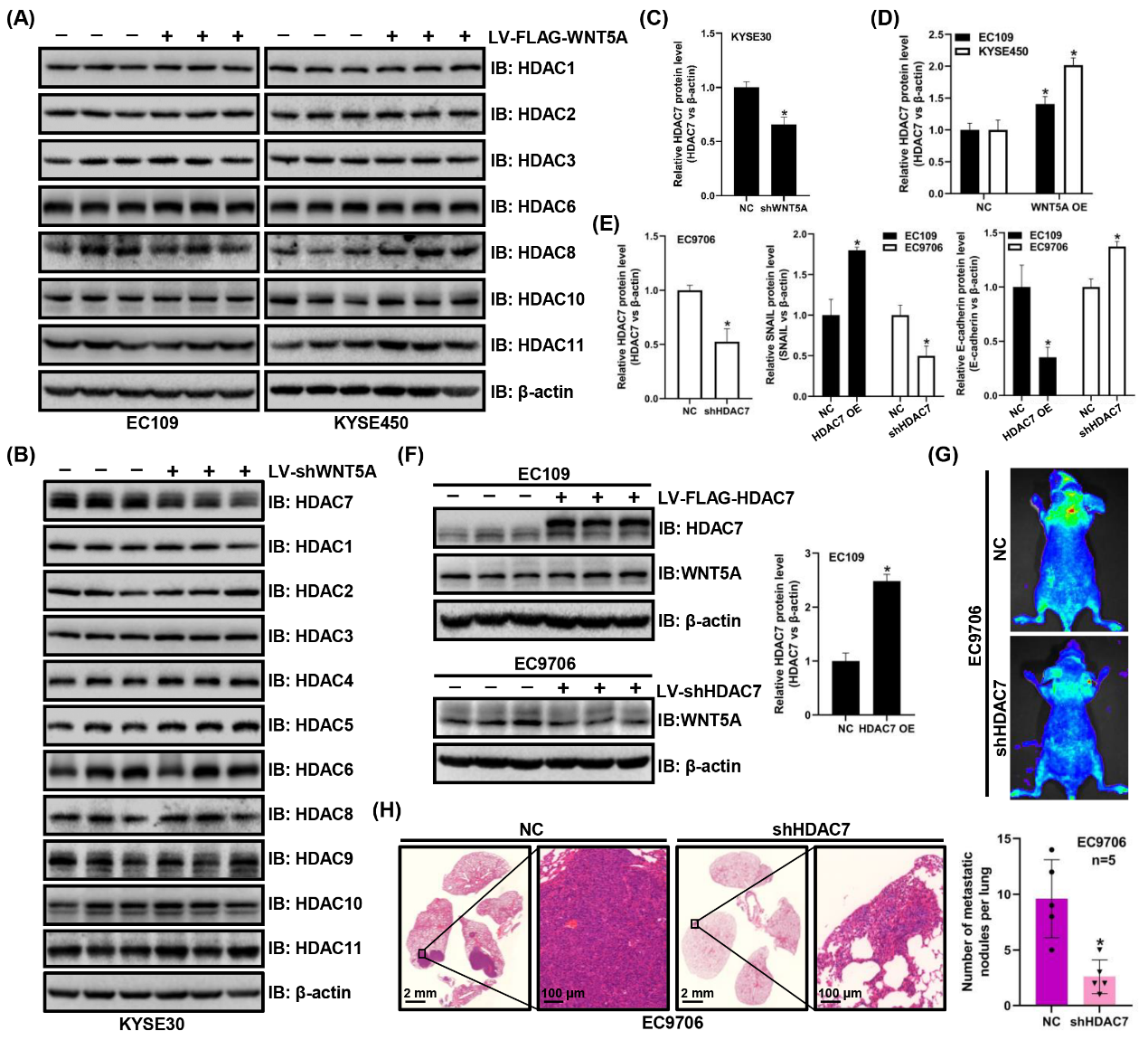


**Supplementary Fig. 2** (A) Western blots showing the levels of HDAC1, HDAC2, HDAC3, HDAC6, HDAC8, HDAC10, and HDAC11 in WNT5A-overexpressing ESCC cells. (B-C) Western blot showing HDAC1-11 levels in WNT5A knockdown KYSE30 cells. (D) Statistical analysis of the HDAC7 level based on the western blotting results presented in Figure 4A. (E) Statistical analysis of the HDAC7, SNAIL, and E-cadherin levels based on the western blotting results presented in Figure 4C. (F) Western blot showing the levels of WNT5A and HDAC7 in HDAC7-overexpressing or HDAC7 knockdown ESCC cells. (G) Representative fluorescence images of nude mice 7 weeks after the tail vein injection. (H) Representative images of HE staining of lung samples and statistical analysis of the metastatic nodules per lung (n=5 per group). Scale bars, 2 mm and 100 μm (inset). β-actin was used as an internal control in western blot assays. **P* < 0.05 compared with the NC group. Abbreviation: LV, lentivirus.


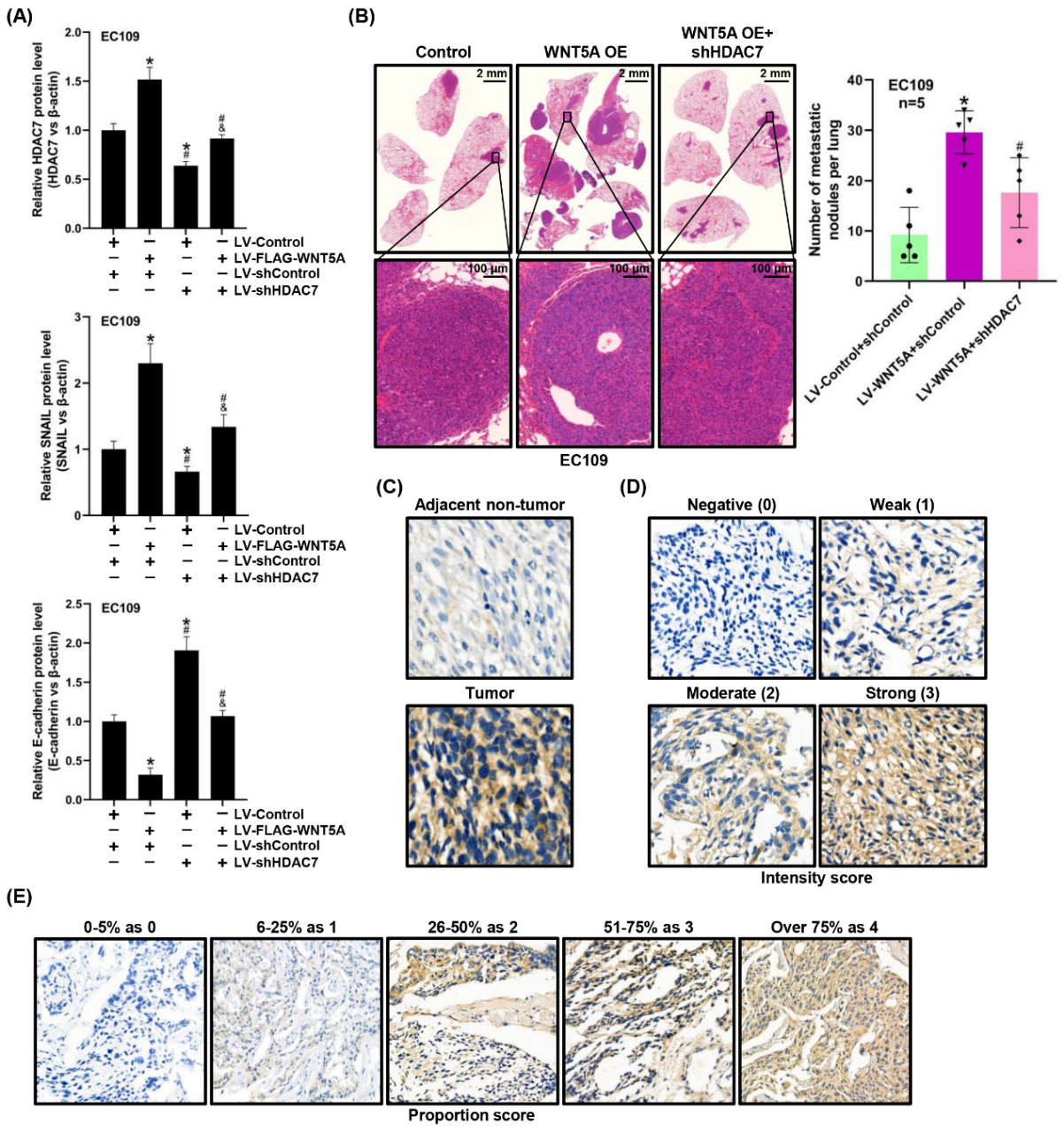


**Supplementary Fig. 3** (A) Statistical analysis of HDAC7, SNAIL, and E-cadherin levels based on the western blotting results presented in Figure 5A. **P* < 0.05 compared with the LV-Control+shControl group, ^#^*P* < 0.05 compared with the LV-FLAG-WNT5A+shControl group, and ^&^*P* < 0.05 compared with the LV-Control+shHDAC7 group. (B) Representative images of HE staining of lung samples and statistical analysis of the metastatic nodules per lung from the indicated groups (n=5 per group). Scale bars, 2 mm and 100 μm (inset). **P* < 0.05 compared with the Control group and ^#^*P* < 0.05 compared with the WNT5A OE group. (C) Representative IHC images of ESCC tissues and adjacent nontumor tissues. (D) Representative IHC images showing various expression intensities in ESCC tissues. (E) Representative IHC images showing various expression proportions in ESCC tissues.

**Supplementary Table 1. Univariate analysis of the correlations between clinicopathological variables and the survival of patients with ESCC.**

| **Clinicopathological variables** | **Cumulative**  **survival rates (%)** | | **Median survival time (month)** | **Univariate analysis** | | |
| --- | --- | --- | --- | --- | --- | --- |
|  | **3-Years** | **5-Years** |  | **HR** | **95% CI** | ***P* value** |
| **Age** |  |  |  | 1.251 | 0.806-1.943 | 0.317 |
| <60 | 51.1±7.5 | 44.4±7.4 | 41±10.730 |  |  |  |
| ≥60 | 40.0±4.9 | 35.0±4.8 | 27±4.999 |  |  |  |
| **Gender** |  |  |  | 1.057 | 0.626-1.785 | 0.835 |
| Female | 42.3±9.7 | 42.3±9.7 | 32±8.286 |  |  |  |
| Male | 43.7±4.5 | 37.0±4.4 | 29±3.776 |  |  |  |
| **Smoking history** |  |  |  | 1.106 | 0.736-1.664 | 0.628 |
| Never | 45.6±6.6 | 42.1±6.5 | 32±6.470 |  |  |  |
| Ever | 42.0±5.3 | 35.2±5.1 | 27+5.542 |  |  |  |
| **Tumor size** |  |  |  | 1.229 | 0.802-1.884 | 0.344 |
| <5 cm | 46.9±7.1 | 40.8±7.0 | 35±8.165 |  |  |  |
| ≥5 cm | 41.7±5.0 | 36.5±4.9 | 26±4.899 |  |  |  |
| **TNM stages** |  |  |  | 1.805 | 1.203-2.708 | 0.004 |
| I-II | 57.7±5.9 | 50.7±5.9 | 63±18.000 |  |  |  |
| III-IV | 29.7±5.3 | 25.7±5.1 | 18±4.301 |  |  |  |
| **Tumor invasion** |  |  |  | 1.17 | 0.567-2.412 | 0.671 |
| T1-T2 | 46.2±13.8 | 46.2±13.8 | 34±24.715 |  |  |  |
| T3-T4 | 43.2±4.3 | 37.1±4.2 | 29±3.590 |  |  |  |
| **Lymphatic invasion** |  |  |  | 1.874 | 1.253-2.803 | 0.002 |
| N0 | 57.7±5.6 | 51.3±5.7 | 63±16.579 |  |  |  |
| N1-N3 | 26.9±5.4 | 22.4±5.1 | 18+3.508 |  |  |  |
| **Differentiation** |  |  |  | 1.084 | 0.578-2.032 | 0.802 |
| Well and moderate | 43.0±4.4 | 37.5±4.3 | 31±3.536 |  |  |  |
| Poorly and not | 47.1±12.1 | 41.2±11.9 | 27±21.952 |  |  |  |
| **WNT5A expression** |  |  |  | 3.986 | 2.576-6.166 | <0.001 |
| Low | 70.4±5.4 | 62.0±5.8 | 89±NA |  |  |  |
| High | 17.6±4.4 | 14.9±4.1 | 15±1.955 |  |  |  |
| **SNAIL expression** |  |  |  | 5.311 | 3.343-8.436 | <0.001 |
| Low | 74.3±5.2 | 65.7±5.7 | 90±NA |  |  |  |
| High | 14.7±4.1 | 12.0±3.8 | 14±2.163 |  |  |  |

**Supplementary Table 2. Multivariate analysis of the correlations between the clinicopathological variables of patients with ESCC.**

| **Clinicopathological variables** | **Multivariate analysis** | | |
| --- | --- | --- | --- |
|  | **HR** | **95% CI** | ***P* value** |
| TNM stages | 1.905 | 1.142-3.176 | 0.013 |
| Lymphatic invasion | 0.626 | 0.307-1.275 | 0.197 |
| Tumor WNT5A expression | 2.310 | 1.423-3.748 | 0.001 |
| Tumor SNAIL expression | 3.596 | 2.145-6.030 | <0.001 |

**Supplementary Table 3.** Detailed information on the LV-WNT5A and LV-HADC7 lentiviruses.

| No. | Accession No | Vector name | Titer (TU/ml) | Antibiotic resistance |
| --- | --- | --- | --- | --- |
| LV-WNT5A (63407-1) | NM_003392 | pGC-FU-3FLAG-SV40-EGFP-IRES-Hygromycin | 2×10^8^ | hygromycin |
| LV-HDAC7（40995-4） | NM_001098416 | pGC-FU-3FLAG-CBh-gcGFP-IRES-puromycin | 2×10^8^ | puromycin |

**Supplementary Table 4.** Detailed information on the LV-shWNT5A and LV-shHADC7 lentiviruses.

| No. | Accession No | Vector name | Target Seq | Titer (TU/ml) | Antibiotic resistance |
| --- | --- | --- | --- | --- | --- |
| WNT5A-RNAi(25532-1) | NM_003392 | pFU-GW-007 | caCATGCAGTACATCGGAGAA | 7×10^8^ | puromycin |
| HDAC7-  RNAi (78750-1) | NM_015401 | pFU-GW-007 | gcCAGCAAGATCCTCATTGTA | 9×10^8^ | puromycin |
