# Supplementary figures and images for: WNT5A promotes the metastasis of esophageal squamous cell carcinoma by activating the HDAC7/SNAIL signaling pathway
Source: Cell Death Dis. 2022 May 20;13(5):480. doi: 10.1038/s41419-022-04901-x (PMC9122958; doi:10.1038/s41419-022-04901-x)

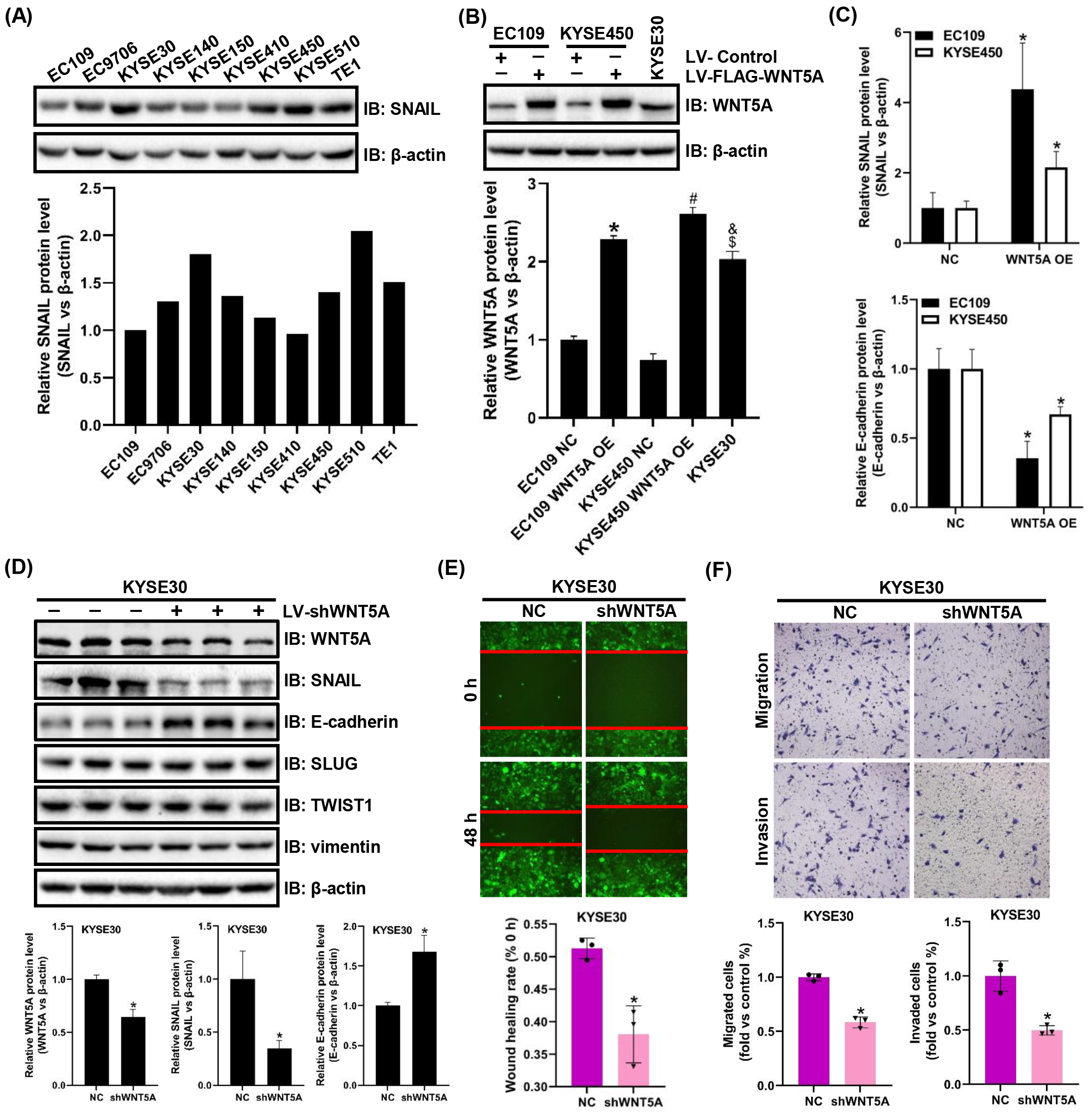

Supplement: Supplementary file 2 — Supplementary Figure 1 [file 41419_2022_4901_MOESM2_ESM.tif]

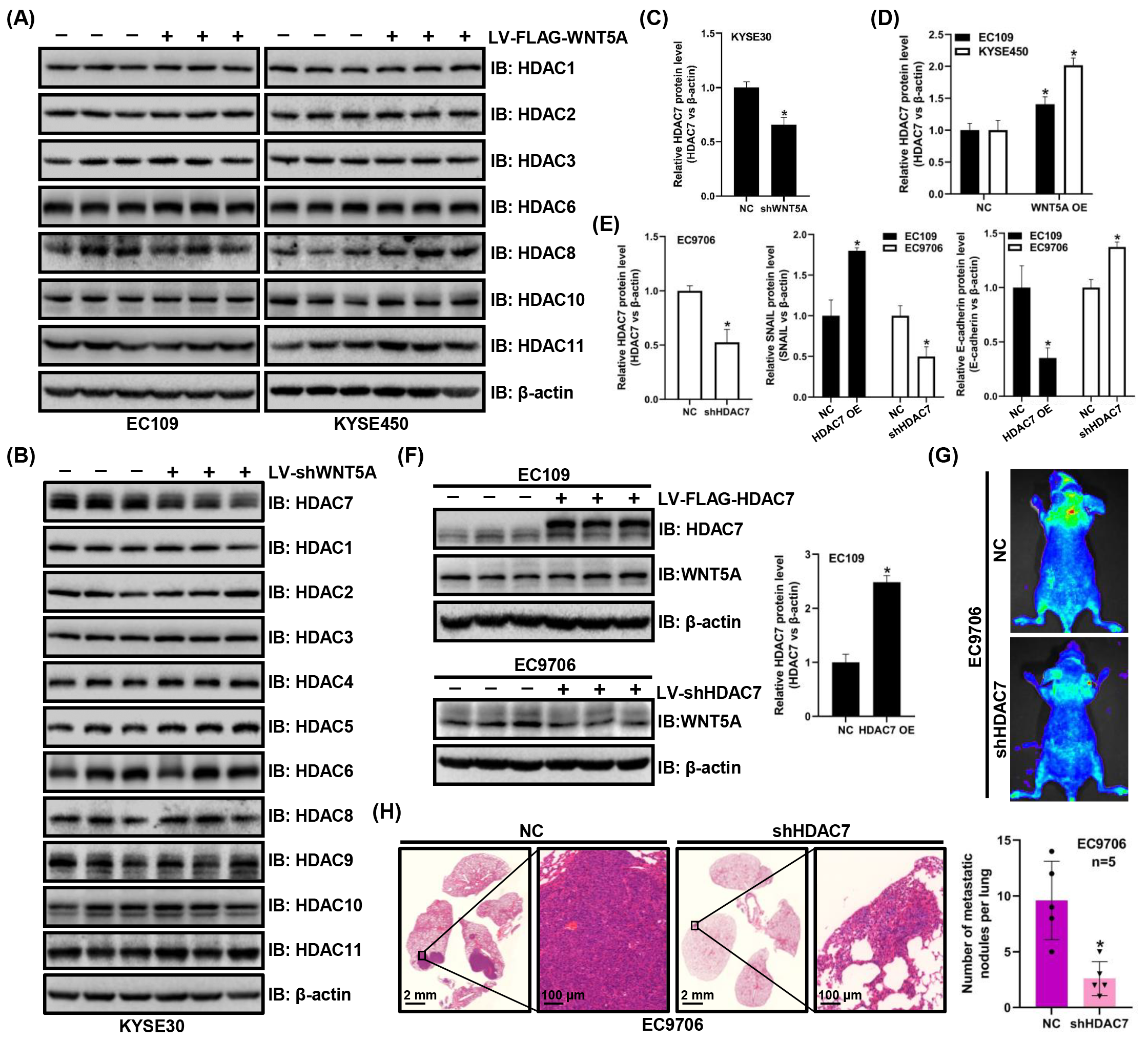

Supplement: Supplementary file 3 — Supplementary Figure 2 [file 41419_2022_4901_MOESM3_ESM.tif]

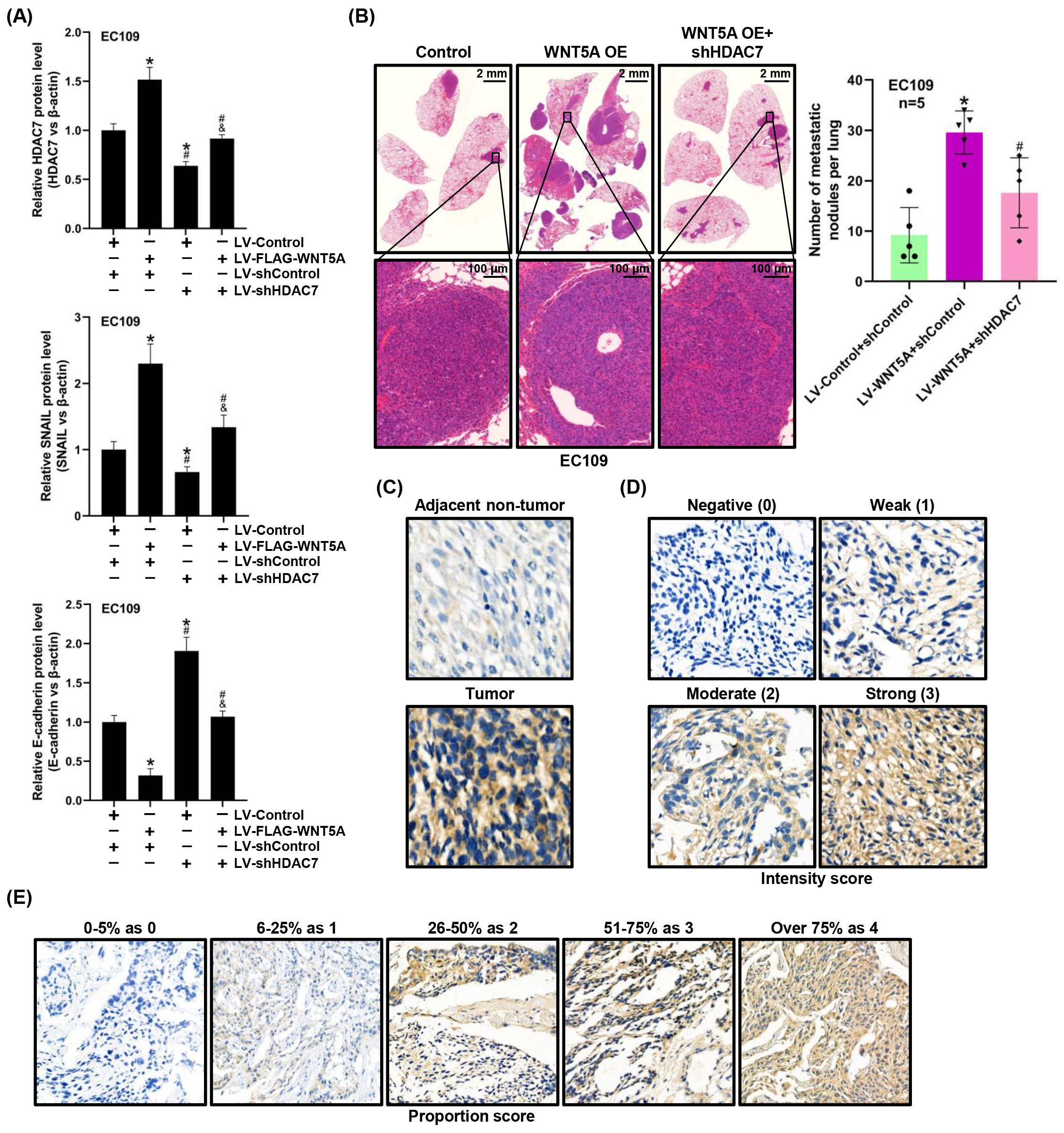

Supplement: Supplementary file 4 — Supplementary Figure 3 [file 41419_2022_4901_MOESM4_ESM.tif]

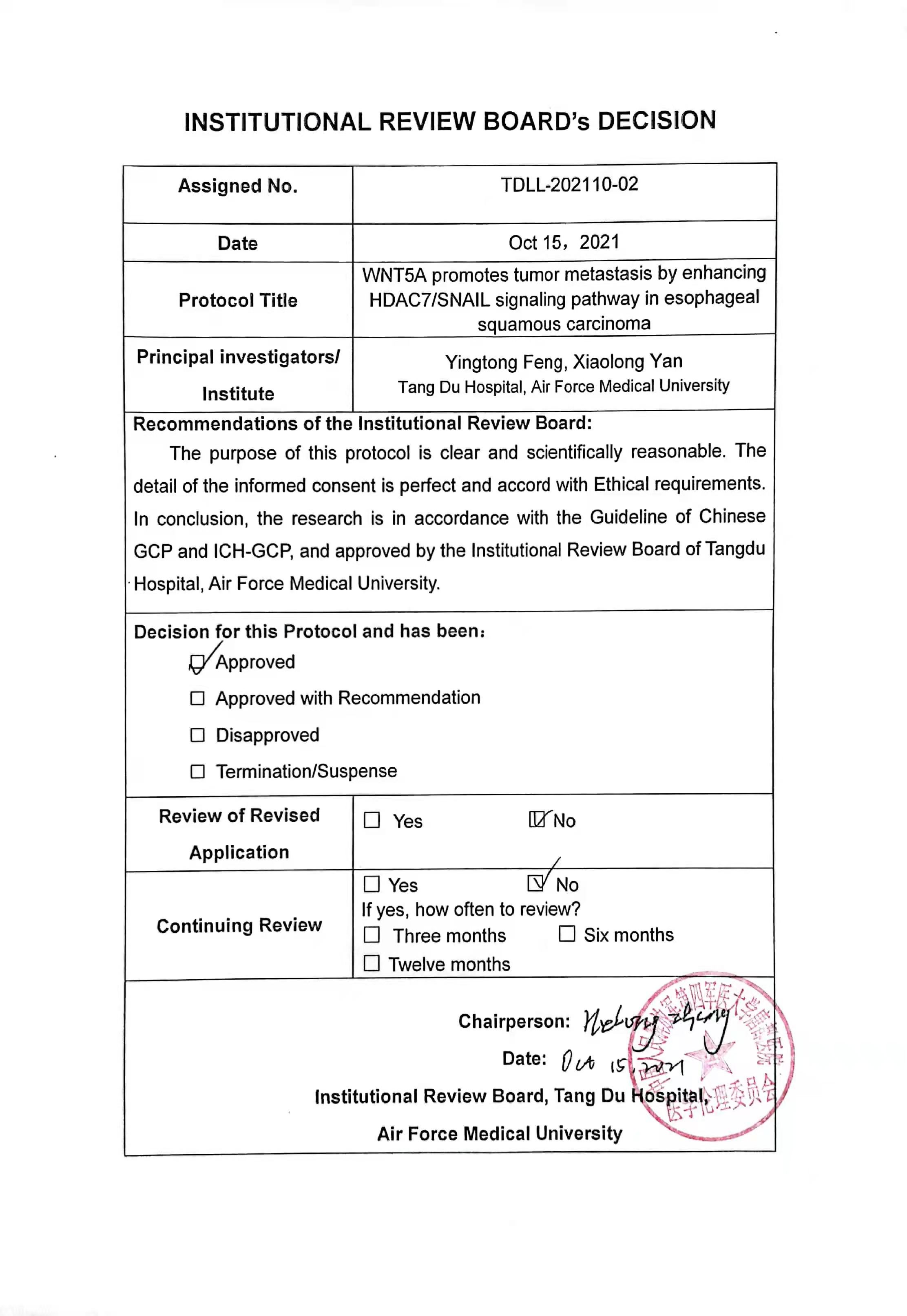

Supplement: Supplementary file 10 — Clinical research ethics review form [file 41419_2022_4901_MOESM10_ESM.jpg]
