# Supplementary material for: WNT5A promotes the metastasis of esophageal squamous cell carcinoma by activating the HDAC7/SNAIL signaling pathway
Source: Cell Death Dis. 2022 May 20;13(5):480. doi: 10.1038/s41419-022-04901-x (PMC9122958; doi:10.1038/s41419-022-04901-x)
Supplement: Supplementary file 5 — Supplementary Table 1 [file 41419_2022_4901_MOESM5_ESM.docx]

**Supplementary Table 1. Univariate analysis of the correlations between clinicopathological variables and the survival of patients with ESCC.**

| **Clinicopathological variables** | **Cumulative**  **survival rates (%)** | | **Median survival time (month)** | **Univariate analysis** | | |
| --- | --- | --- | --- | --- | --- | --- |
|  | **3-Years** | **5-Years** |  | **HR** | **95% CI** | ***P* value** |
| **Age** |  |  |  | 1.251 | 0.806-1.943 | 0.317 |
| <60 | 51.1±7.5 | 44.4±7.4 | 41±10.730 |  |  |  |
| ≥60 | 40.0±4.9 | 35.0±4.8 | 27±4.999 |  |  |  |
| **Gender** |  |  |  | 1.057 | 0.626-1.785 | 0.835 |
| Female | 42.3±9.7 | 42.3±9.7 | 32±8.286 |  |  |  |
| Male | 43.7±4.5 | 37.0±4.4 | 29±3.776 |  |  |  |
| **Smoking history** |  |  |  | 1.106 | 0.736-1.664 | 0.628 |
| Never | 45.6±6.6 | 42.1±6.5 | 32±6.470 |  |  |  |
| Ever | 42.0±5.3 | 35.2±5.1 | 27+5.542 |  |  |  |
| **Tumor size** |  |  |  | 1.229 | 0.802-1.884 | 0.344 |
| <5 cm | 46.9±7.1 | 40.8±7.0 | 35±8.165 |  |  |  |
| ≥5 cm | 41.7±5.0 | 36.5±4.9 | 26±4.899 |  |  |  |
| **TNM stages** |  |  |  | 1.805 | 1.203-2.708 | 0.004 |
| I-II | 57.7±5.9 | 50.7±5.9 | 63±18.000 |  |  |  |
| III-IV | 29.7±5.3 | 25.7±5.1 | 18±4.301 |  |  |  |
| **Tumor invasion** |  |  |  | 1.17 | 0.567-2.412 | 0.671 |
| T1-T2 | 46.2±13.8 | 46.2±13.8 | 34±24.715 |  |  |  |
| T3-T4 | 43.2±4.3 | 37.1±4.2 | 29±3.590 |  |  |  |
| **Lymphatic invasion** |  |  |  | 1.874 | 1.253-2.803 | 0.002 |
| N0 | 57.7±5.6 | 51.3±5.7 | 63±16.579 |  |  |  |
| N1-N3 | 26.9±5.4 | 22.4±5.1 | 18+3.508 |  |  |  |
| **Differentiation** |  |  |  | 1.084 | 0.578-2.032 | 0.802 |
| Well and moderate | 43.0±4.4 | 37.5±4.3 | 31±3.536 |  |  |  |
| Poorly and not | 47.1±12.1 | 41.2±11.9 | 27±21.952 |  |  |  |
| **WNT5A expression** |  |  |  | 3.986 | 2.576-6.166 | <0.001 |
| Low | 70.4±5.4 | 62.0±5.8 | 89±NA |  |  |  |
| High | 17.6±4.4 | 14.9±4.1 | 15±1.955 |  |  |  |
| **SNAIL expression** |  |  |  | 5.311 | 3.343-8.436 | <0.001 |
| Low | 74.3±5.2 | 65.7±5.7 | 90±NA |  |  |  |
| High | 14.7±4.1 | 12.0±3.8 | 14±2.163 |  |  |  |
