# Supplementary material for: WNT5A promotes the metastasis of esophageal squamous cell carcinoma by activating the HDAC7/SNAIL signaling pathway
Source: Cell Death Dis. 2022 May 20;13(5):480. doi: 10.1038/s41419-022-04901-x (PMC9122958; doi:10.1038/s41419-022-04901-x)
Supplement: Supplementary file 6 — Supplementary Table 2 [file 41419_2022_4901_MOESM6_ESM.docx]

**Supplementary Table 2. Multivariate analysis of the correlations between the clinicopathological variables of patients with ESCC.**

| **Clinicopathological variables** | **Multivariate analysis** | | |
| --- | --- | --- | --- |
|  | **HR** | **95% CI** | ***P* value** |
| TNM stages | 1.905 | 1.142-3.176 | 0.013 |
| Lymphatic invasion | 0.626 | 0.307-1.275 | 0.197 |
| Tumor WNT5A expression | 2.310 | 1.423-3.748 | 0.001 |
| Tumor SNAIL expression | 3.596 | 2.145-6.030 | <0.001 |
