# Supplementary material for: WNT5A promotes the metastasis of esophageal squamous cell carcinoma by activating the HDAC7/SNAIL signaling pathway
Source: Cell Death Dis. 2022 May 20;13(5):480. doi: 10.1038/s41419-022-04901-x (PMC9122958; doi:10.1038/s41419-022-04901-x)
Supplement: Supplementary file 7 — Supplementary Table 3. [file 41419_2022_4901_MOESM7_ESM.docx]

**Supplementary Table 3.** Detailed information on the LV-WNT5A and LV-HADC7 lentiviruses.

| No. | Accession No | Vector name | Titer (TU/ml) | Antibiotic resistance |
| --- | --- | --- | --- | --- |
| LV-WNT5A (63407-1) | NM_003392 | pGC-FU-3FLAG-SV40-EGFP-IRES-Hygromycin | 2×10^8^ | hygromycin |
| LV-HDAC7（40995-4） | NM_001098416 | pGC-FU-3FLAG-CBh-gcGFP-IRES-puromycin | 2×10^8^ | puromycin |
