# Supplementary material for: WNT5A promotes the metastasis of esophageal squamous cell carcinoma by activating the HDAC7/SNAIL signaling pathway
Source: Cell Death Dis. 2022 May 20;13(5):480. doi: 10.1038/s41419-022-04901-x (PMC9122958; doi:10.1038/s41419-022-04901-x)
Supplement: Supplementary file 8 — Supplementary Table 4 [file 41419_2022_4901_MOESM8_ESM.docx]

**Supplementary Table 4.** Detailed information on the LV-shWNT5A and LV-shHADC7 lentiviruses.

| No. | Accession No | Vector name | Target Seq | Titer (TU/ml) | Antibiotic resistance |
| --- | --- | --- | --- | --- | --- |
| WNT5A-RNAi(25532-1) | NM_003392 | pFU-GW-007 | caCATGCAGTACATCGGAGAA | 7×10^8^ | puromycin |
| HDAC7-  RNAi (78750-1) | NM_015401 | pFU-GW-007 | gcCAGCAAGATCCTCATTGTA | 9×10^8^ | puromycin |
