# Supplementary material for: WNT5A promotes the metastasis of esophageal squamous cell carcinoma by activating the HDAC7/SNAIL signaling pathway
Source: Cell Death Dis. 2022 May 20;13(5):480. doi: 10.1038/s41419-022-04901-x (PMC9122958; doi:10.1038/s41419-022-04901-x)
Supplement: Supplementary file 11 — The tab of animal experimental ethical inspection [file 41419_2022_4901_MOESM11_ESM.pdf]

动物实验福利与伦理审查表（附表）

编号(No)：IACUC-20210390

项目名称：WNT5A通过上调HDAC7/SNAIL促进食管鳞癌转移的研究
WNT5A promotes tumor metastasis by enhancing the HDAC7/SNAIL signaling pathway in esophageal squamous cell carcinoma

项目类别：国家自然科学基金

动物实验过程及结果概述：(包括动物实验方法、观测指标、安乐死方式等。)

动物实验设施条件是否与拟开展动物实验的规范性要求相匹配的描述:
动物实验方法：将肿瘤细胞悬液经鼠尾静脉注射入裸鼠，每只注射体积不超过0.1ml，根据分组情况进行必要的药物处理，每三天称量裸鼠体重，小动物成像观察肺转移瘤变化，适时取出肺组织行进一步组织学检测。
观测指标：观测不同组别之间肺转移瘤数量及体积。
安乐死方式：吸入式气体麻醉。

动物实验项目的目的、必要性、意义和如何设计以达成研究目标的:
目的：通过观察对照组与不同实验组之间肺转移瘤的体积，明确不同组别细胞的转移能力变化。
必要性：裸鼠肺转移瘤等动物试验为肿瘤表型实验必不可少的一部分，可显著增强文章论证水平及层次。
意义：在体水平明确目的基因对于肿瘤进展的影响。
如何达成研究目标：在前期细胞实验的基础上，进一步进行此处动物实验，两个水平相互验证，明确目的基因对于肿瘤进展的影响。

选择实验动物种类和数量的原因:
裸鼠：裸鼠是肿瘤实验中最稳定的动物模型鼠。本实验需研究不同基因过表达及敲低及加用药物处理后对肿瘤进展的影响，共需12组，每组6只，共72只。

详细列出对动物可能造成的所有可预期的伤害，包括动物运输、每个实验方案动物饲养方式、实验操作步骤中等可能产生伤害或不适的细节以及拟采取的防控措施:
1. 注射过程中可能造成的伤口感染:我们严格遵守无菌原则，多次消毒
2. 在取皮下瘤时造成的疼痛及伤害：我们使用气体麻醉的方法，降低这一部分伤害。

主要观察指标:
1. 每三天检测裸鼠体重。
2. 适时小动物成像观察肺转移瘤变化。

仁慈终点或实验终结的指标:
在肺转移瘤模型成功后5-7周，我们将终止实验

动物处死方法:
气体吸入麻醉处死

动物替代、减少动物用量、降低动物痛苦伤害的主要措施:
1. 严格计算动物使用数量，不浪费实验动物
2. 皮下注射，取材等均对动物进行麻醉及消毒，减轻动物疼痛及可能的伤害。

是否使用有毒（害）物质（感染、放射、化学毒、其他）:
否

利害分析的小结，说明为何预期的利益多余害处:
本动物实验是课题的在体实验部分，增加了本课题的说理层次及证据等级。此外，在动物使用中，我们严格控制数量，并尽可能的减轻动物痛苦及伤害。

信息公开和保密要求，说明哪些信息需要保密，哪些信息可以公开:
均可公开

动物实验伦理审查表

The Tab of Animal Experimental Ethical Inspection

编号(Nọ)：IACUC-20210590

申请人填写的相关信息 (Concerned information wrote by applicant)

申请单位(Name of organization)：唐都医院胸腔外科

申请人学历 (Education of applicant)	博士生	技术职称 Professional title)	中级	岗位证书编号 (Number of permit)	

实验名称(Study title)：裸鼠经鼠尾静脉肺转移瘤及皮下成瘤实验

经费来源(Funding source)：国家自然科学基金面上项目（81870866）

动物情况

动物来源(Source of animal)：动物中心购买	
品种品系(Species or strain)：裸鼠	等级(Grade)：SPF
数量(Number)：72只(♀72只；♂00只)	申请日期(Application date)：2021年03月01日
进驻日期(Entering date)：2021年03月03日	结束日期(Ending date)：2022年02月10日

实验要点，包括实验方法、观测指标、实验结束后处死动物的方法等(Outline of experiments, experimental methods, observational index, executing animal method et. al)：

实验方法：将肿瘤细胞悬液经鼠尾静脉注射入裸鼠，每只注射体积不超过0.1ml，根据分组情况进行必要的药物处理，每三天称量裸鼠体重，小动物成像观察肺转移瘤变化，适时取出肺组织行进一步组织学检测。

观测指标：观测不同组别之间肺转移瘤数量及体积。

动物处死方法：使用吸入麻醉方法进行麻醉。

申请人(Signature of applicant)：冯英同	联系电话(Telephone)：15950675891

审查依据 (Inspection contents)

该项目是否必须用实验动物进行实验，即能否用计算机模拟、细胞培养等非生命方法替代动物或用低等动物替代高等动物进行实验(Does laboratory animal must be used in the project? Could other methods such as computer simulation, cell cultivation or using the low-grade animal instead of the high-grade animal)?

表中所填申请人资格和所用动物的品种品系、质量等级、规格是否合适，能否通过改良设计方案或用高质量的动物来减少所用动物的数量(Are the qualification of applicant, species or strain, grade and specifications of animals suitable? Could the quantity of animals be reduced by improving the study design or using high quality animals)?

能否通过改进实验方法、调整实验观测指标、改良处死动物的方法，来优化实验方案、善待动物(Could the Study design and animal treatment be refined by ameliorating experimental method, adjusting observational index, executing animal method)?

审查结果 (是否同意实验方案)(Results of inspection)

课题负责人意见 (Study director)	☑ 同意(Agree)	☐ 不同意(Disagree)	签名(Signature) 闫小龙
动物实验部意见(Director of Dept. of Lab Animal Science)	☑ 同意(Agree)	☐ 不同意(Disagree)	签名(Signature) [illegible]
实验动物福利与伦理 委员会 (Laboratory Animal Welfare and Ethics Committee)	☑ 同意(Agree)	☐ 不同意(Disagree)	签章(Stamp) 第四军医大学实验动物中心 福利与伦理委员会

备注(Supplement)：
