# Supplementary material for: WNT5A promotes the metastasis of esophageal squamous cell carcinoma by activating the HDAC7/SNAIL signaling pathway
Source: Cell Death Dis. 2022 May 20;13(5):480. doi: 10.1038/s41419-022-04901-x (PMC9122958; doi:10.1038/s41419-022-04901-x)

Figure 2 (A)

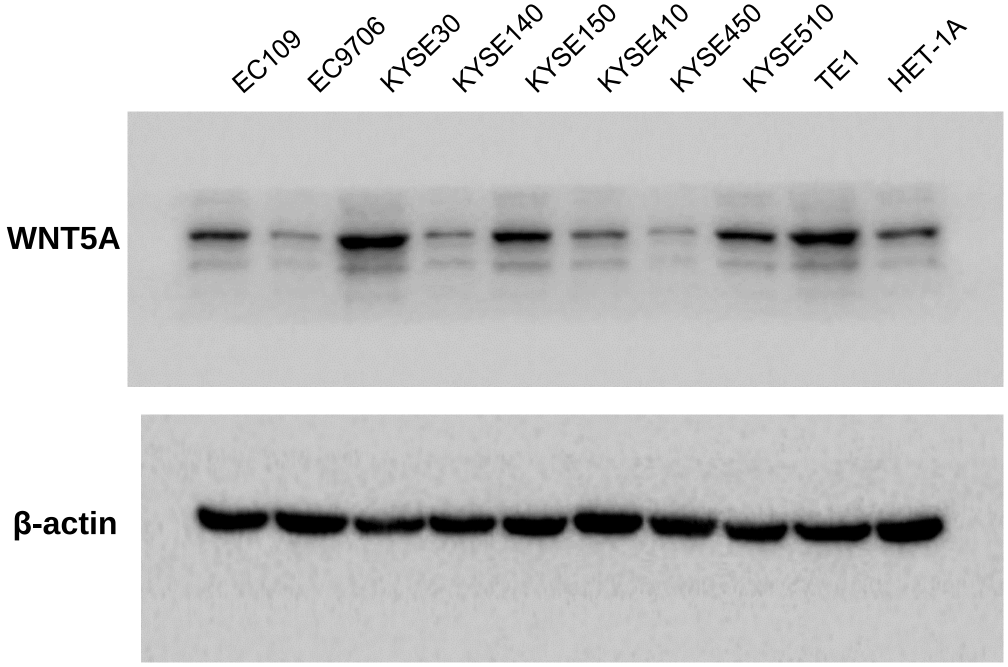
EC109
EC9706
KYSE30
KYSE140
KYSE150
KYSE410
KYSE450
KYSE510
TE1
HET-1A
WNT5A
β-actin

Figure 2 (B)

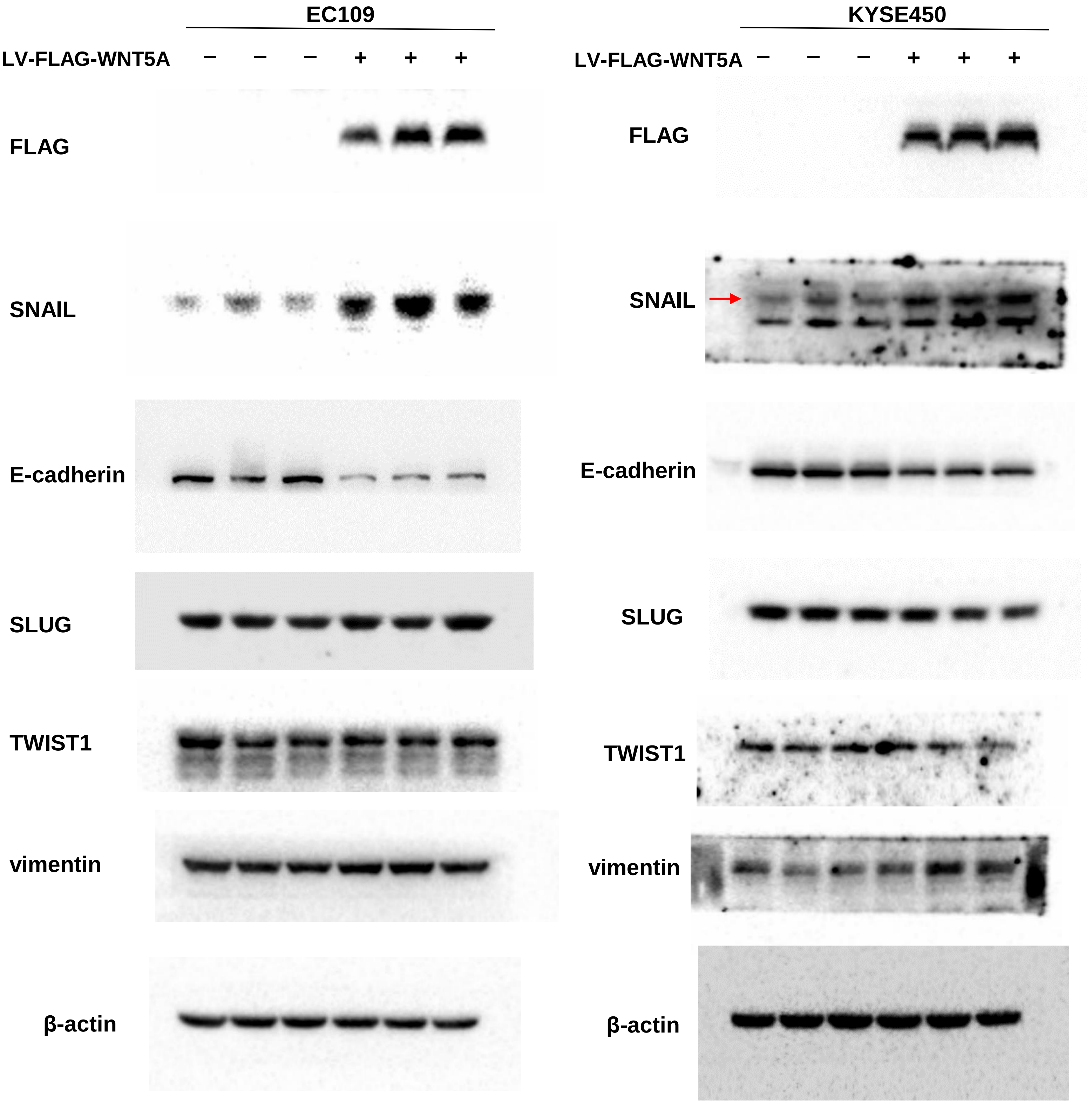
EC109
KYSE450
LV-FLAG-WNT5A − − − + + +
LV-FLAG-WNT5A − − − + + +
FLAG
SNAIL
E-cadherin
SLUG
TWIST1
vimentin
β-actin

Figure 4 (A)

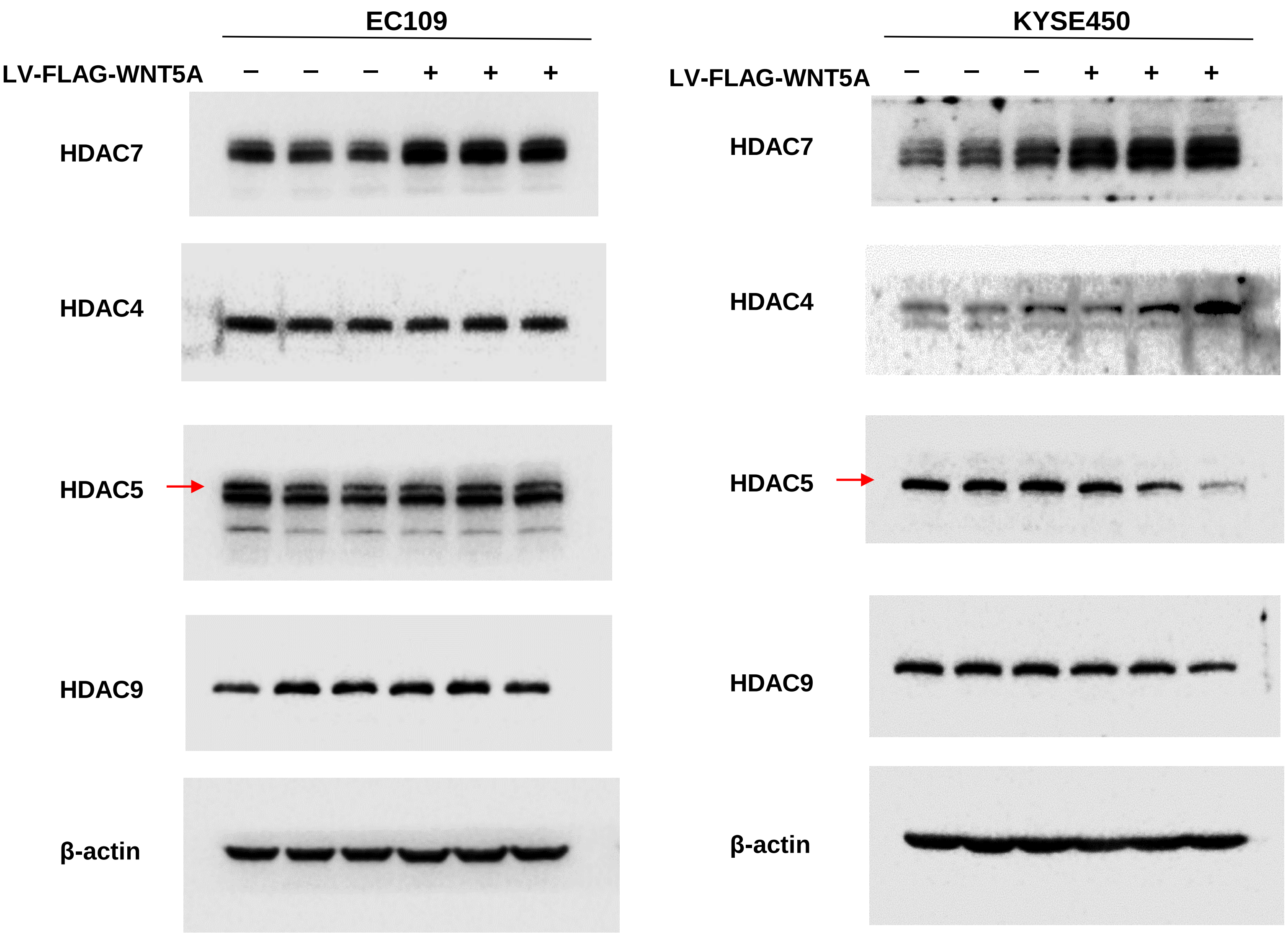
EC109
LV-FLAG-WNT5A − − − + + +
HDAC7
HDAC4
HDAC5
HDAC9
β-actin
KYSE450
LV-FLAG-WNT5A − − − + + +
HDAC7
HDAC4
HDAC5
HDAC9
β-actin

Figure 4 (B)

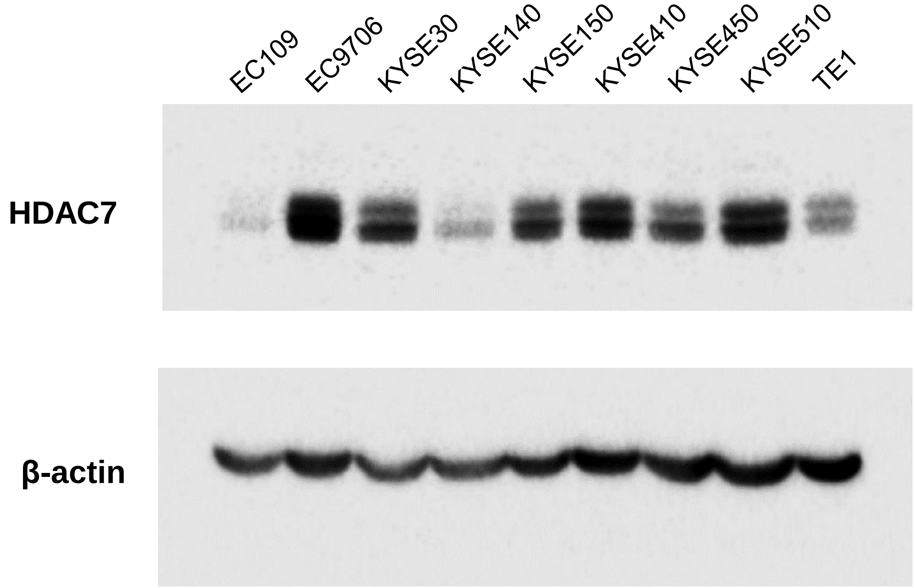
EC109
EC9706
KYSE30
KYSE140
KYSE150
KYSE410
KYSE450
KYSE510
TE1
HDAC7
β-actin

Figure 4 (C)

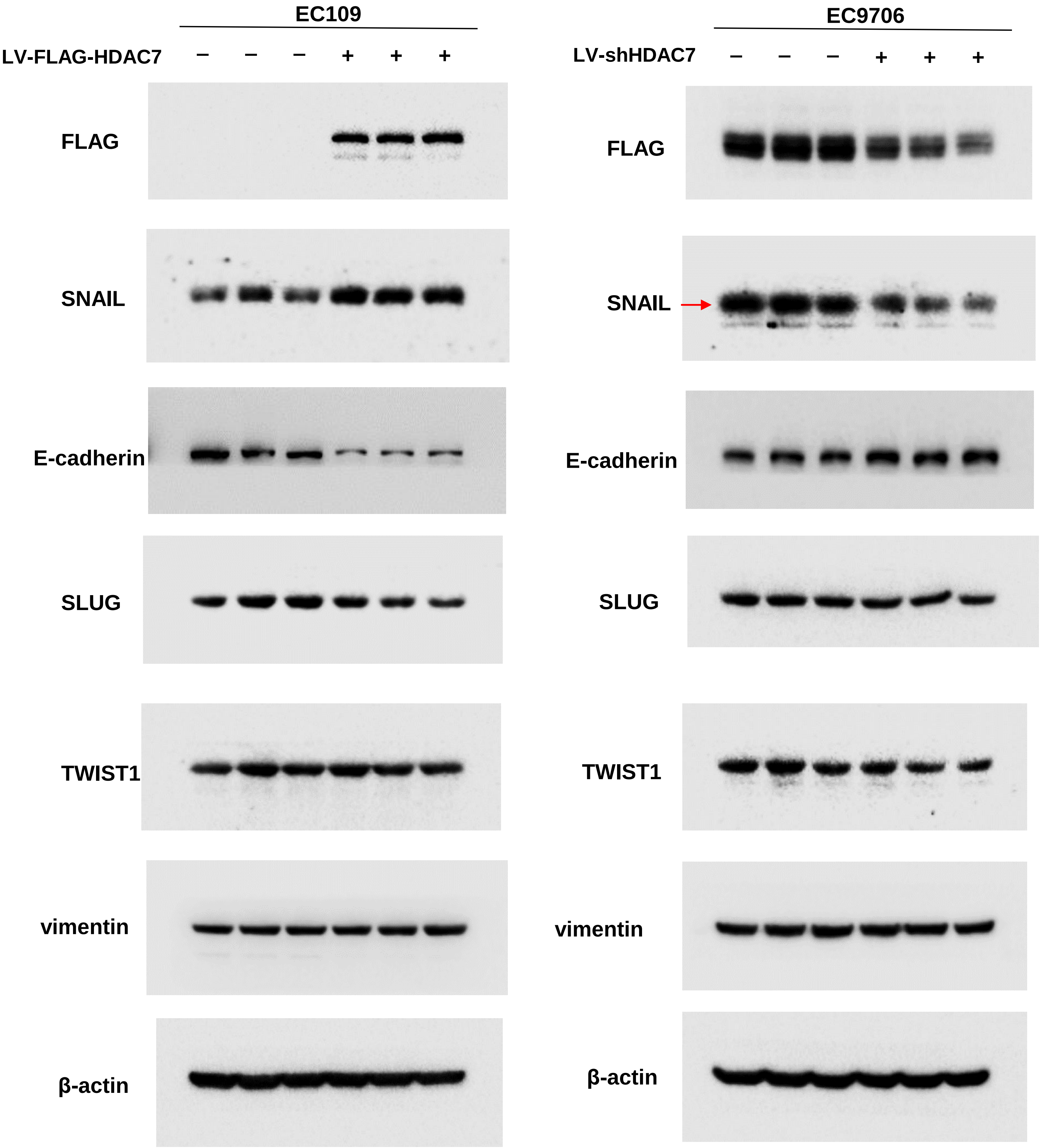
EC109
EC9706
LV-FLAG-HDAC7 − − − + + +
LV-shHDAC7 − − − + + +
FLAG
SNAIL
E-cadherin
SLUG
TWIST1
vimentin
β-actin
FLAG
SNAIL
E-cadherin
SLUG
TWIST1
vimentin
β-actin

Figure5 (A)

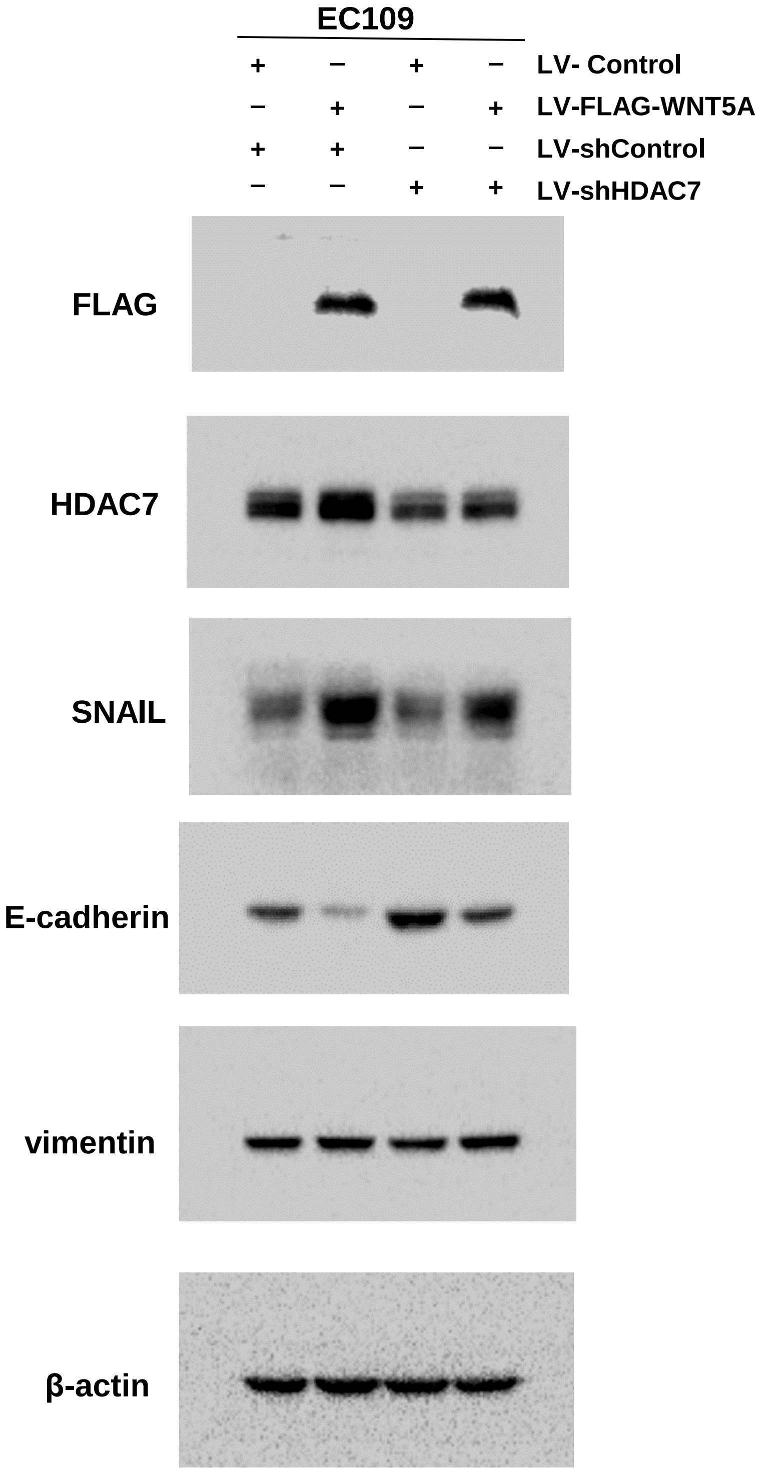

EC109
LV- Control
LV-FLAG-WNT5A
LV-shControl
LV-shHDAC7
FLAG
HDAC7
SNAIL
E-cadherin
vimentin
β-actin

Figure6 (A) -Left

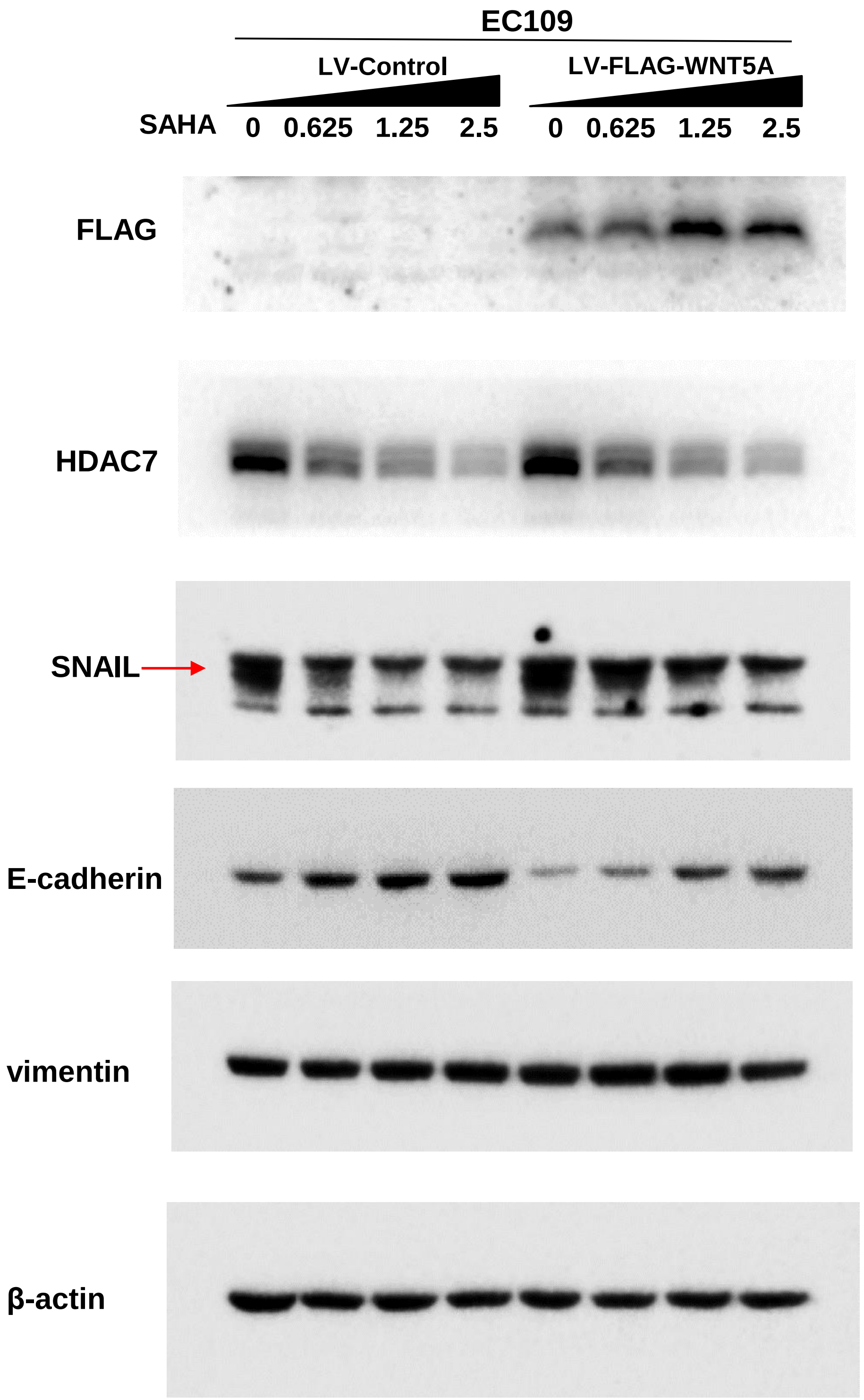

Figure6 (A)-Right

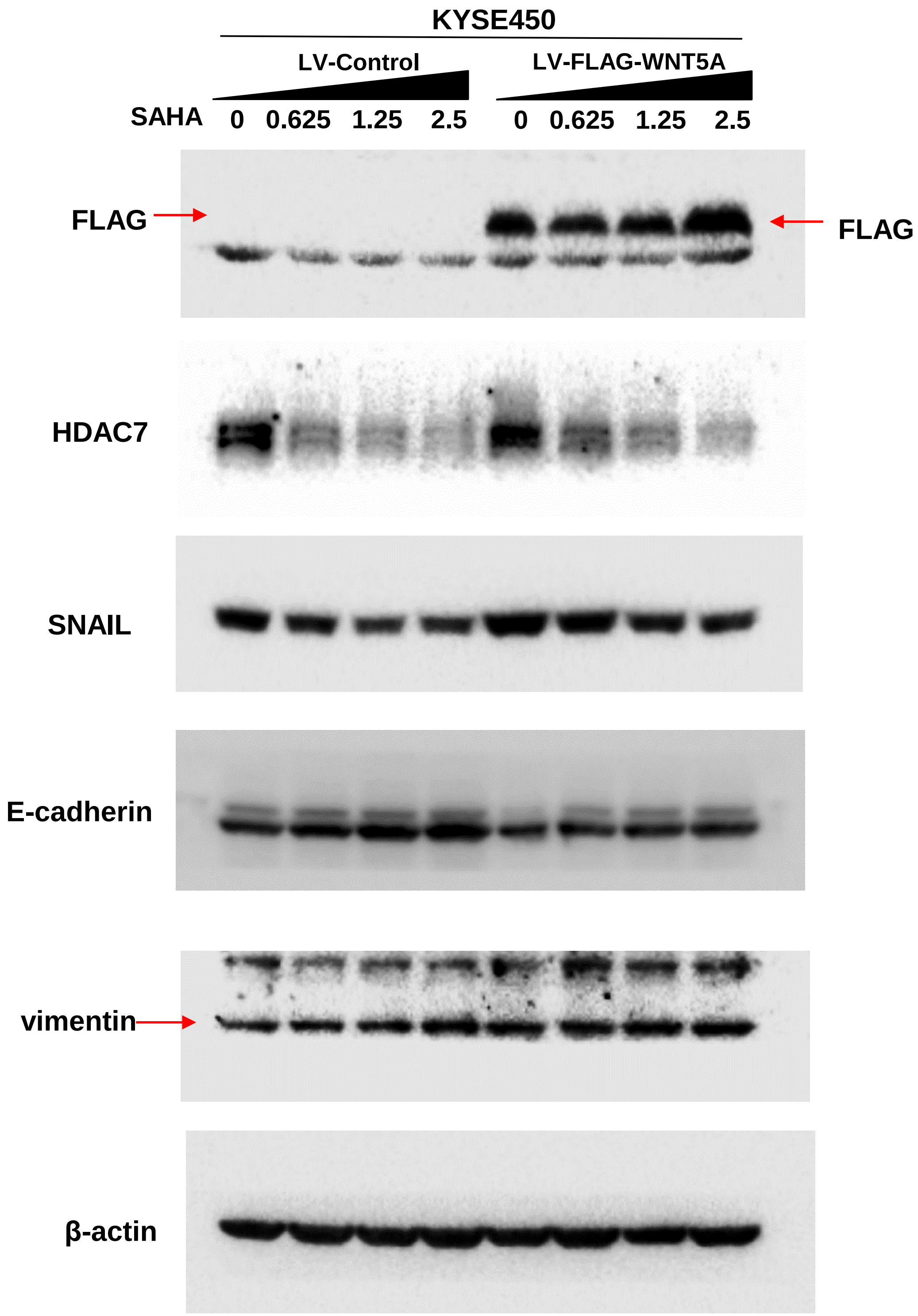

Figure7 (A) -Left

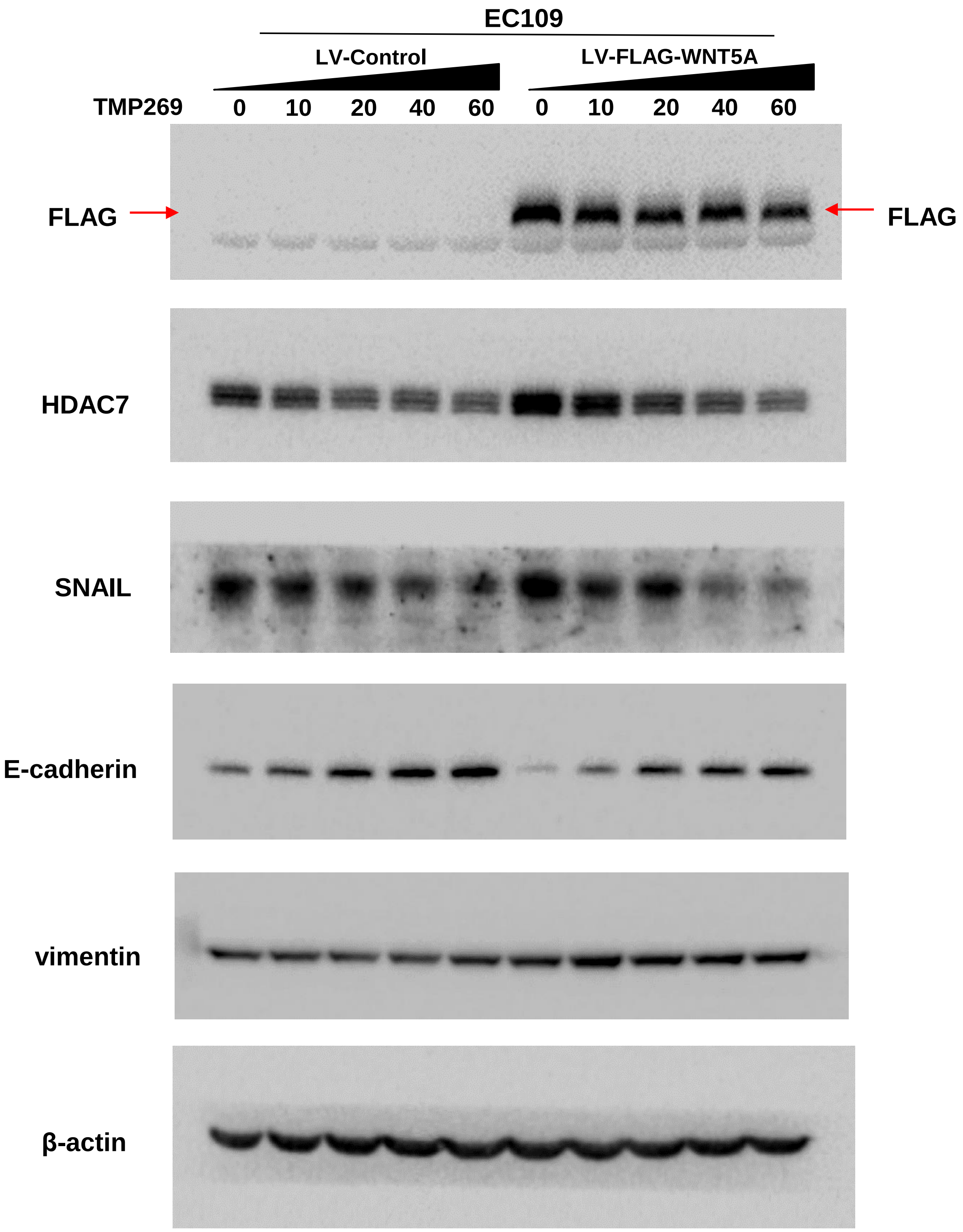

Figure7 (A) -Right

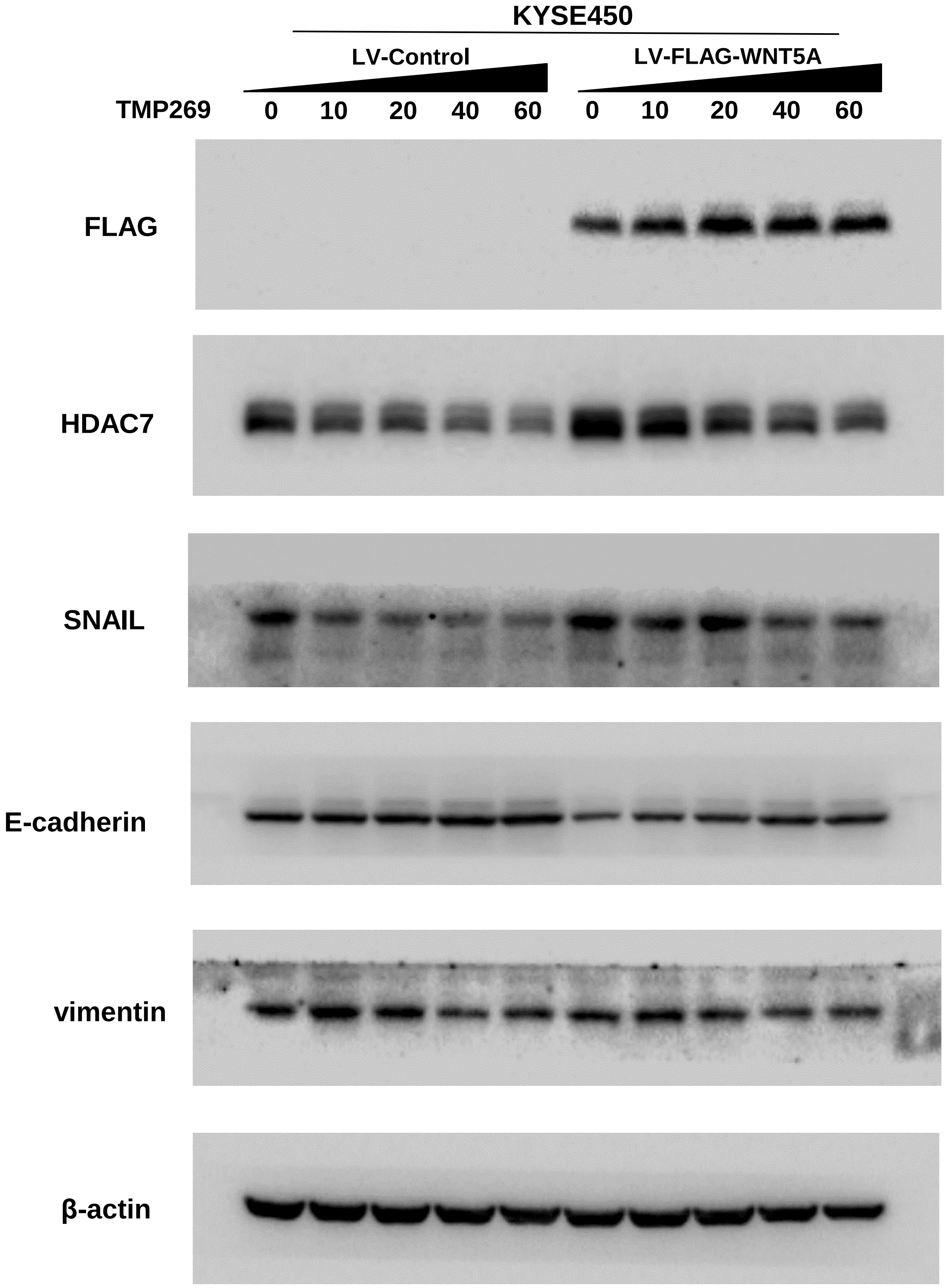

Supplementary figure 1 (A)

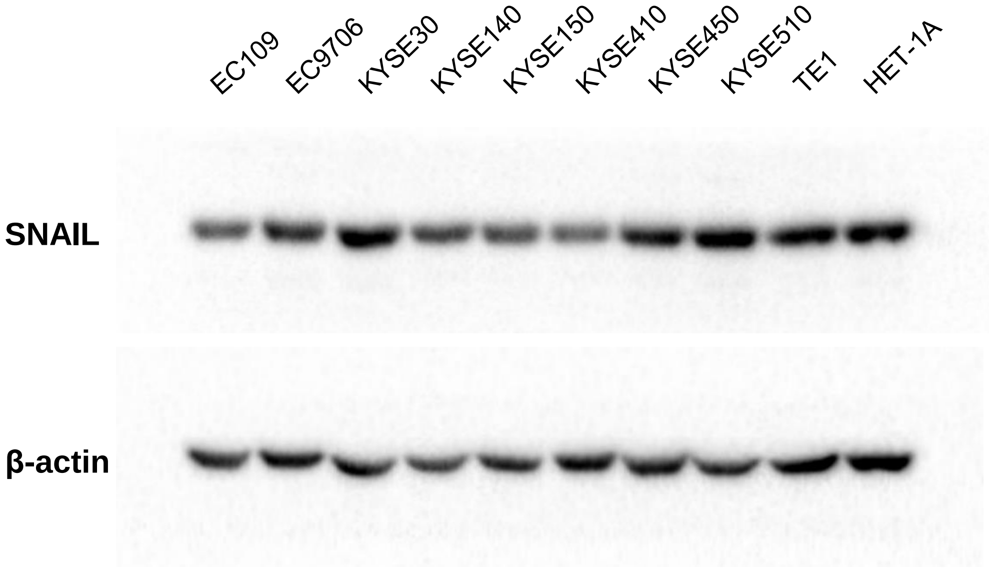

Supplementary figure 1 (B)

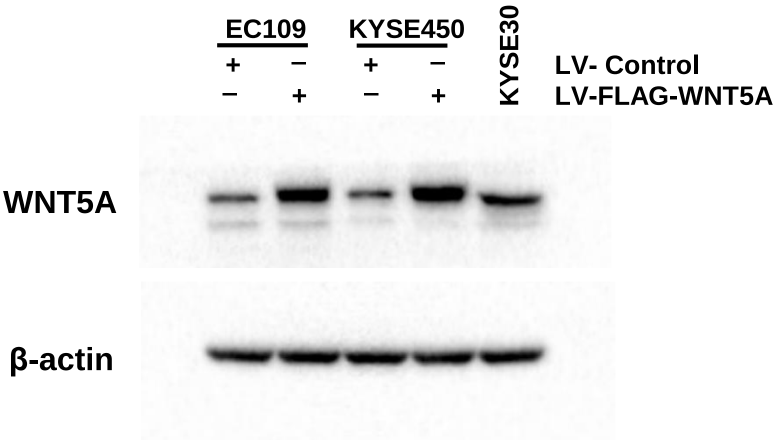

Supplementary figure 1 (C)

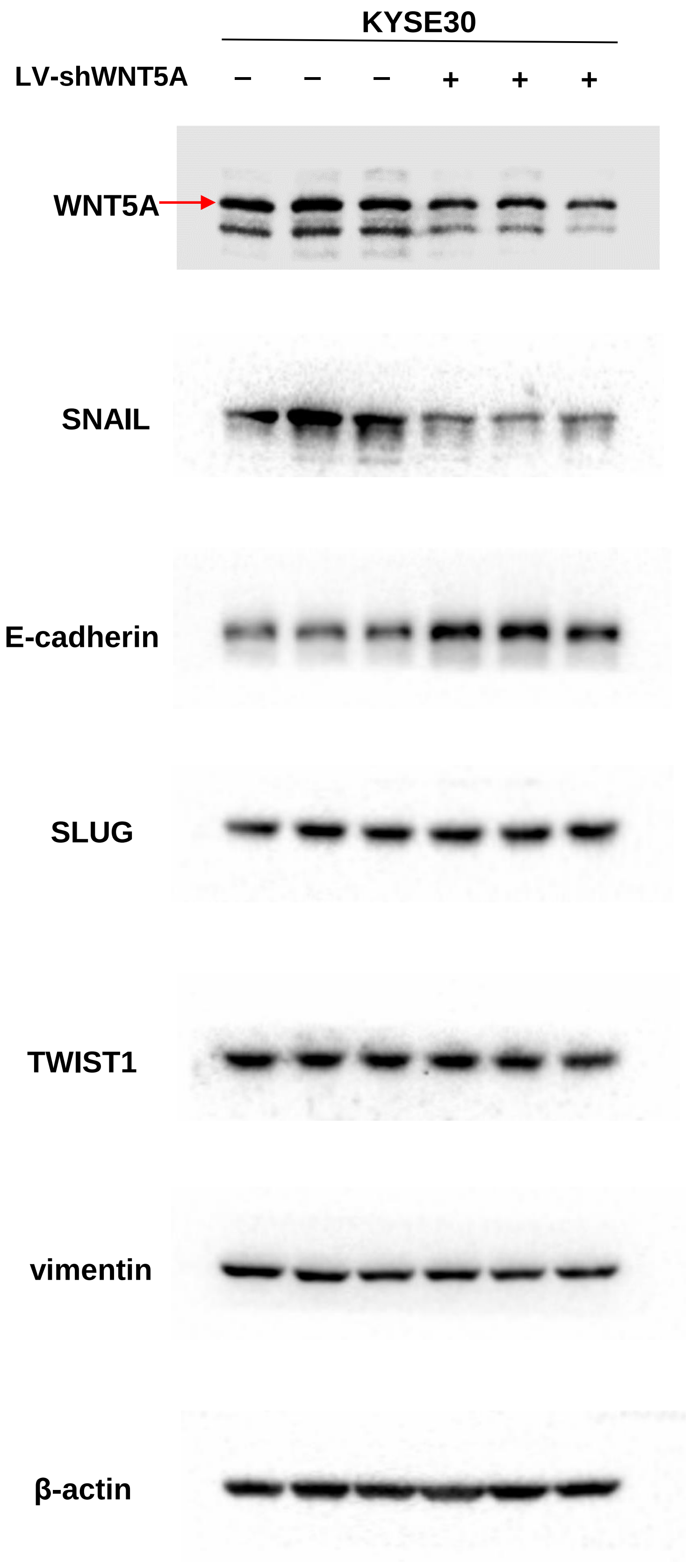

Supplementary figure 2 (A)

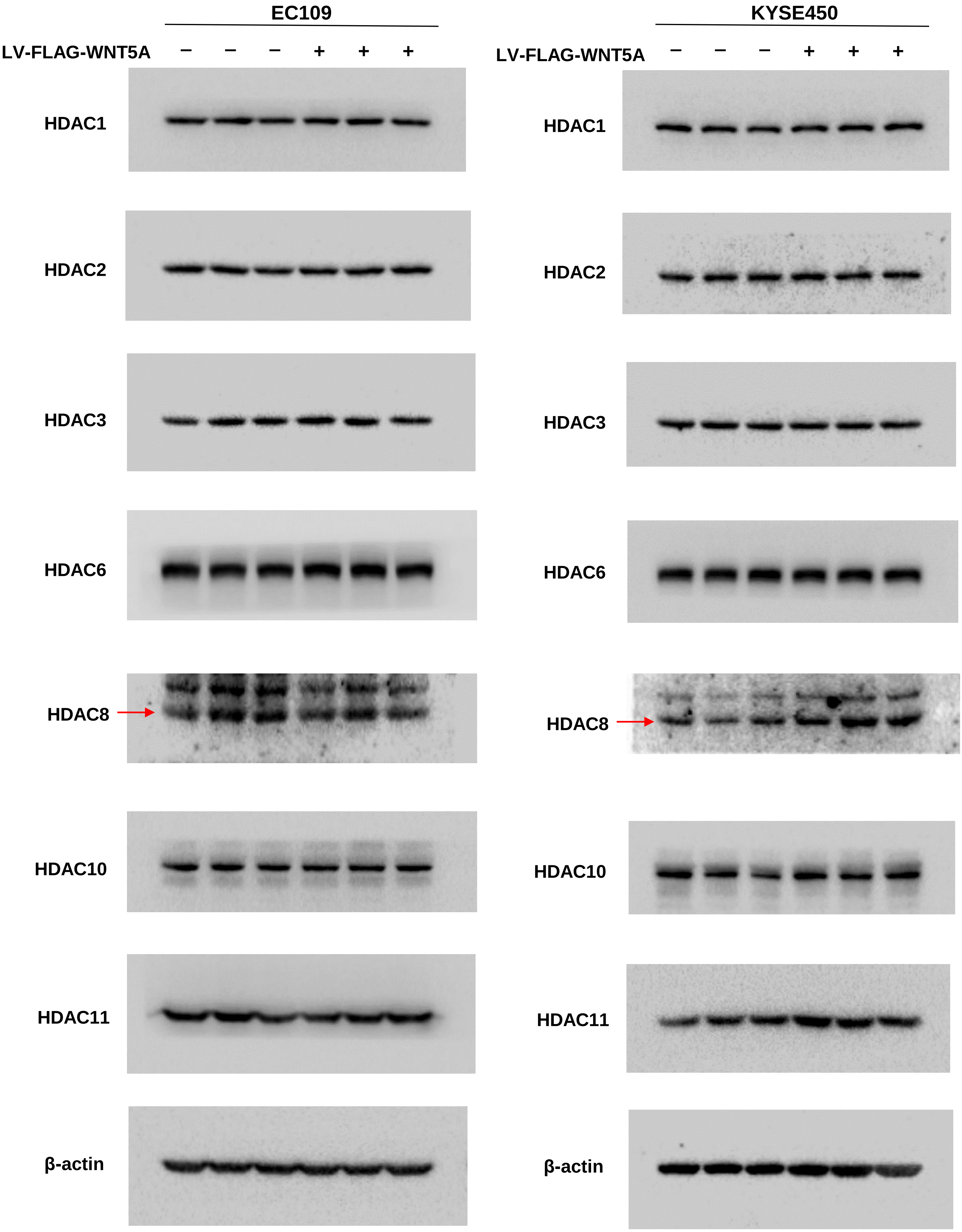

Supplementary figure 2 (B)

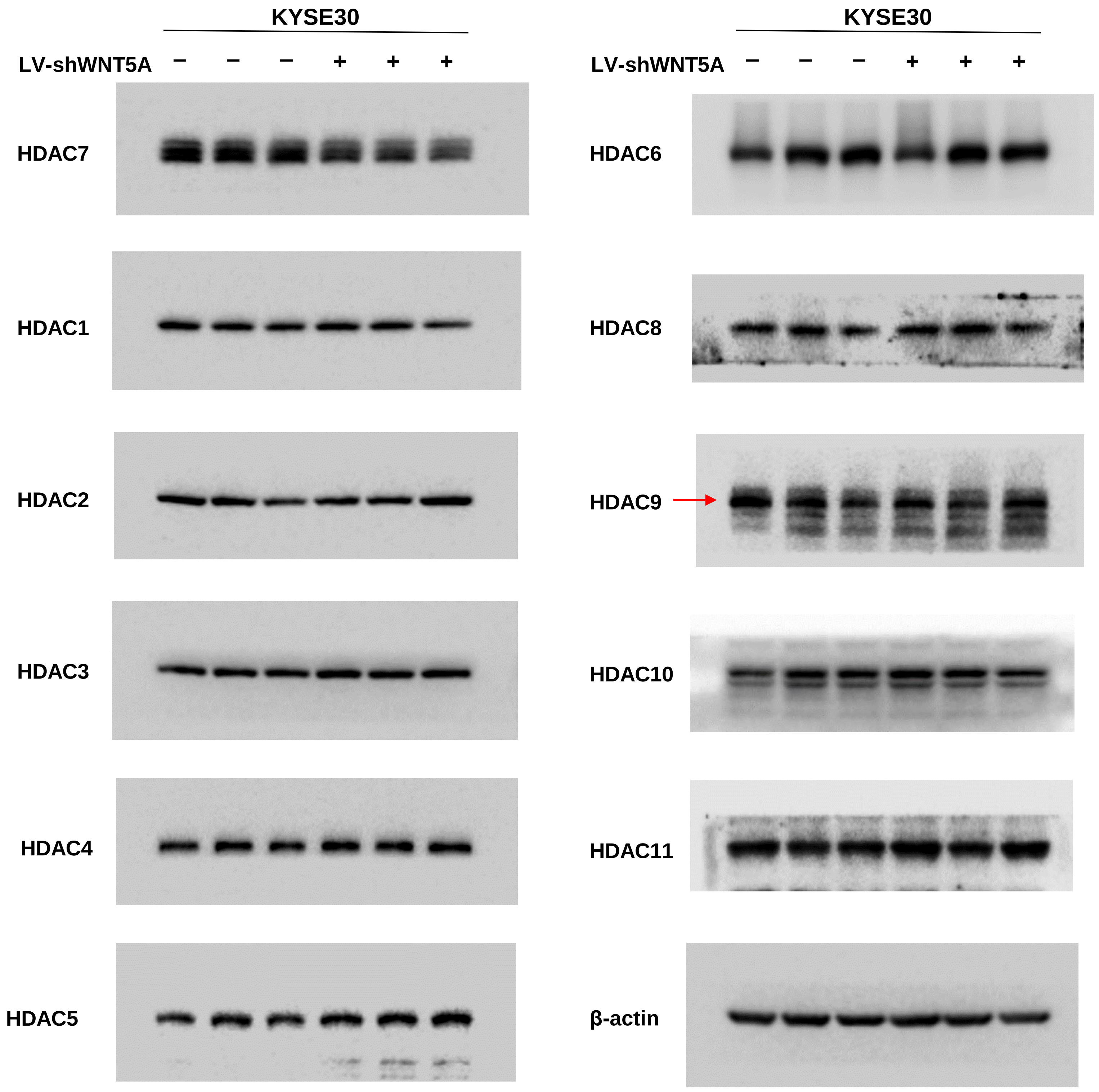

Supplementary figure 2 (F)

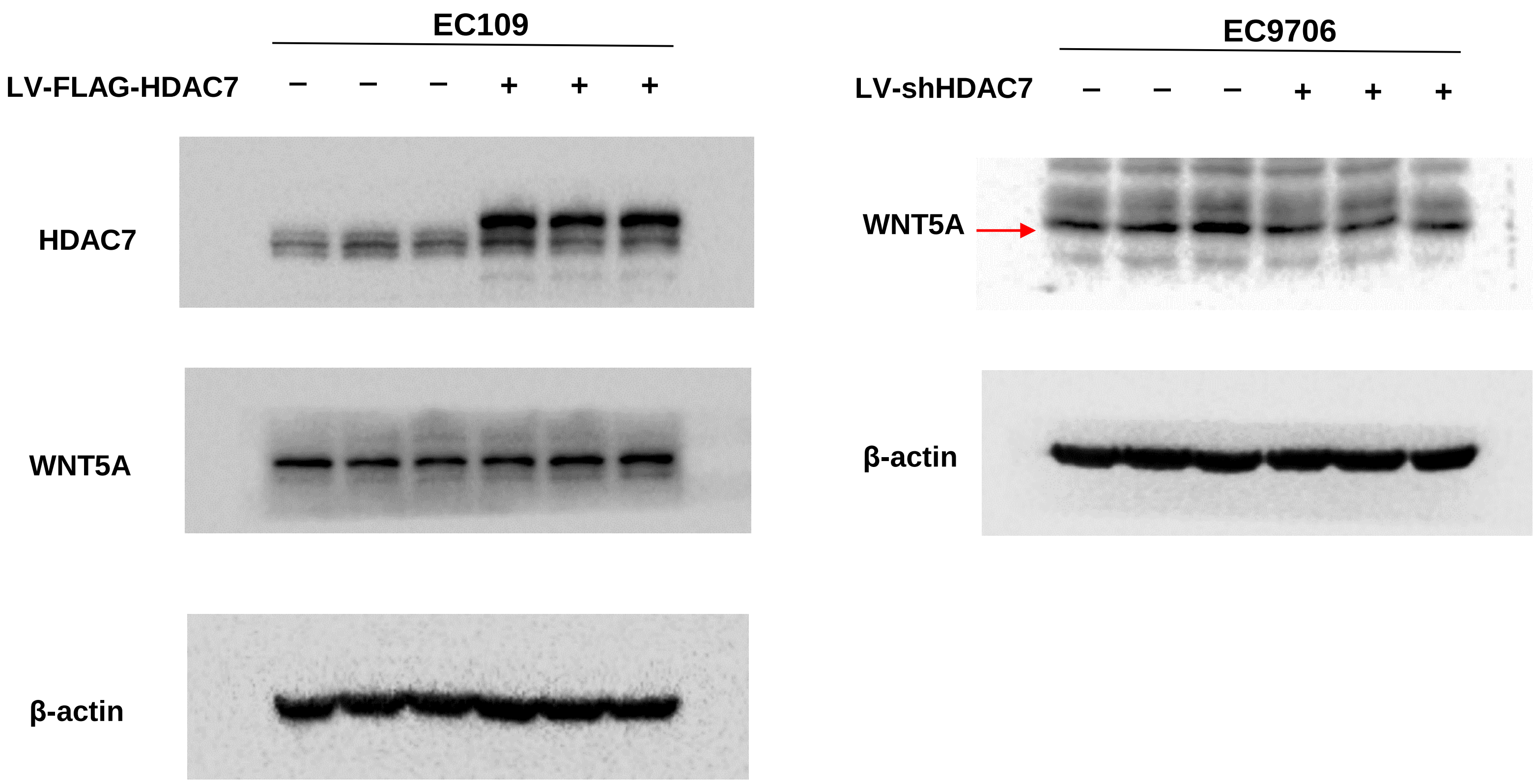

Supplement: Supplementary file 12 — Original Data File [file 41419_2022_4901_MOESM12_ESM.pdf]
